# Spatial and temporal variation of net primary productivity of herbaceous marshes and its climatic drivers in China

**DOI:** 10.3389/fpls.2024.1380081

**Published:** 2024-05-14

**Authors:** Liyuan Wu, Xiangjin Shen, Jiaqi Zhang, Yiwen Liu, Chen Ding, Rong Ma, Xianguo Lu, Ming Jiang

**Affiliations:** ^1^ Northeast Institute of Geography and Agroecology, Chinese Academy of Sciences, Changchun, China; ^2^ University of Chinese Academy of Sciences, Beijing, China; ^3^ College of Forestry, Northeast Forestry University, Harbin, China

**Keywords:** herbaceous marshes, vegetation, NPP, climate change, China

## Abstract

Herbaceous marshes are widely distributed in China and are vital to regional ecological security and sustainable development. Vegetation net primary productivity (NPP) is a vital indicator of vegetation growth. Climatic change can significantly affect NPP, but variations in NPP of herbaceous marsh and their responses to climate change in China remain unclear. Using meteorological data and MODIS NPP data during 2000-2020, this study analyzed the spatial and temporal variations of NPP and their responses to climate change in Chinese herbaceous marshes. We found that the annual NPP of herbaceous marshes in China increased significantly at a rate of 3.34 g C/m^2^/a from 2000 to 2020, with an average value of 336.60 g C/m^2^. The increased annual total precipitation enhanced the national average NPP, whereas annual mean temperature had no significant effect on the national average NPP. Regionally, precipitation had a significant positive effect on the NPP in temperate semi-arid and arid and temperate semi-humid and humid marsh regions. For the first time, we discovered asymmetry effects of daytime and nighttime temperatures on NPP in herbaceous marshes of China. In temperate humid and semi-humid marsh regions, increased summer daytime temperature decreased the NPP while increased summer nighttime temperature increased the NPP. In the Tibetan Plateau, increased autumn daytime temperature, as well as summer daytime and nighttime temperatures could increase the NPP of herbaceous marshes. This study highlights the different influences of seasonal climate change on the NPP of herbaceous marshes in China and indicates that the differential effects of daytime and nighttime temperatures should be considering in simulating the NPP of herbaceous marshes in terrestrial ecosystem models, especially under the background of global asymmetric diurnal warming.

## 1 Introduction

Wetland are a key ecosystem type, accounting for approximately 12%-24% of the world’s terrestrial carbon stocks (Shukla et al., 2019), despite covering only 4%-6% of the global area (Mitra et al., 2005). Marsh is an important type of wetland that performs a critical role in supporting ecological stability, conserving biodiversity, and regulating the carbon cycle (Erwin, 2009; Jimenez et al., 2012; Bhowmik, 2022). Vegetation of marshes is essential for conserving water sources, improving water quality, protecting the marsh ecosystem, and promoting surface energy exchange (Clarkson et al., 2013; Salimi et al., 2021). Vegetation net primary productivity (NPP) is a vital indicator of carbon sequestration of marsh wetland ecosystems (Nayak et al., 2010; Reyer et al., 2014; Hammer and Bastian, 2020). Climate change can markedly affect the NPP of marshes and consequently influence the regional carbon cycle (Wang et al., 2022a). A significant change has occurred in the NPP of marshes (Shen et al., 2022b). The clarification of the response of marsh NPP to climatic change is essential for predicting the global carbon cycle (Wang et al., 2022b). A number of studies have recognized that climatic change affects the NPP of terrestrial ecosystems (Li, 2014; Gang et al., 2017), but relatively few research focus on climate impacts on the NPP of marshes (Hirota et al., 2007). Marshes have special water conditions compared to other terrestrial ecosystems (Shen et al., 2020), and the NPP response to climatic change in marshes may differ from that in other terrestrial ecosystems (Shen et al., 2021a). Analysis of the NPP of marshes can improve our understanding of carbon sequestration of this ecosystem, which is important for predicting the impacts of future climate change and carrying out the adaptive manage of marsh ecosystem.

The marsh area in China is the third largest in the world, with herbaceous marshes being the most widespread (Shen et al., 2021a). The rate of carbon sequestration by herbaceous marsh vegetation is faster than that of other types of marsh vegetation (Zhou et al., 2009). Herbaceous marshes play a critical role in regulating regional carbon cycle (Ye et al., 2022). The NPP of herbaceous marsh is a significant indicator of herbaceous marsh ecosystem functions and capacity for carbon sequestration (Woltz et al., 2023). Understanding the NPP changes and clarifying the response of the NPP of herbaceous marshes to climate change is important for predicting carbon stocks in China. Some researchers have studied the changes in the NPP of marshes and their response to changing climatic conditions (Wang et al., 2022a). Yu et al. (2010) analyzed NPP changes in *Deyeuxia angustifolia*, *Carex lasiocarpa*, and *Carex pseudocuraica* in the Sanjiang Plain marshes and concluded that an increase in temperature would lead to a significant increase in NPP. Luo et al. (2021) estimated the NPP of three typical *Phragmites australis* wetlands in northeast China based on remotely sensed and field data and showed that an increase in precipitation led to an increase in NPP of *Phragmites australis* wetlands. Nevertheless, these studies concentrated on the response of a single species or local-scale marsh NPP to climate change and did not study herbaceous marsh vegetation across China. Herbaceous marsh vegetation response to climatic change varies from region to region (Wang et al., 2021). To better estimate carbon storage and reveal future vegetation dynamics throughout China, it is urgent to understand the temporal and spatial variations of NPP and climatic effects in herbaceous marshes of China.

In the context of global climate change, daytime and nighttime temperatures showed asymmetric (different) warming with a larger warming trend of nighttime temperature than daytime temperature (Shen et al., 2014; Liu et al., 2023c). Interestingly, some studies found different effects of daytime and nighttime temperatures on vegetation coverage of herbaceous marsh in China. For example, Shen et al. (2021b) found that, compared with daytime temperature, growing season nighttime temperature had a larger positive effect on vegetation coverage of herbaceous marsh in the cold Tibet Plateau and Northeast China possibly due to reduced freezing damage. However, Wang et al. (2020) found that increased growing season daytime temperature could reduce marsh vegetation coverage because of enhanced evapotranspiration in the arid Songnen Plain of China. Until recently, however, it was unclear whether nighttime and daytime temperatures have different effects on NPP of herbaceous marshes in different regions of China. To further evaluate the carbon sequestration potential and predict carbon sequestration of Chinese herbaceous marshes, it is urgent to research the response of NPP to nighttime and daytime temperature in China.

Based on the MODIS NPP and observed climate data, this study analyzed temporal and spatial variation in NPP of herbaceous marshes in different regions of China and examined the responses of NPP to temperature (including daytime and nighttime temperature) and precipitation changes from 2000 to 2020. Our study aimed to focus explicitly on the following questions: (1) Is the NPP of herbaceous marshes in China increasing or decreasing during the past two decades? (2) Are there differences in the responses of NPP of herbaceous marshes to climatic change at different regions? (3) Are there differential effects of nighttime and daytime temperatures on NPP of herbaceous marshes in different regions? The findings of this study may contribute to reveal the mechanism of response of herbaceous marsh vegetation to climatic change and provide a scientific basis for us to formulate strategies to enhance the ecological functions of wetlands and manage wetland ecosystems.

## 2 Materials and methods

### 2.1 Study region

**Figure 1:**
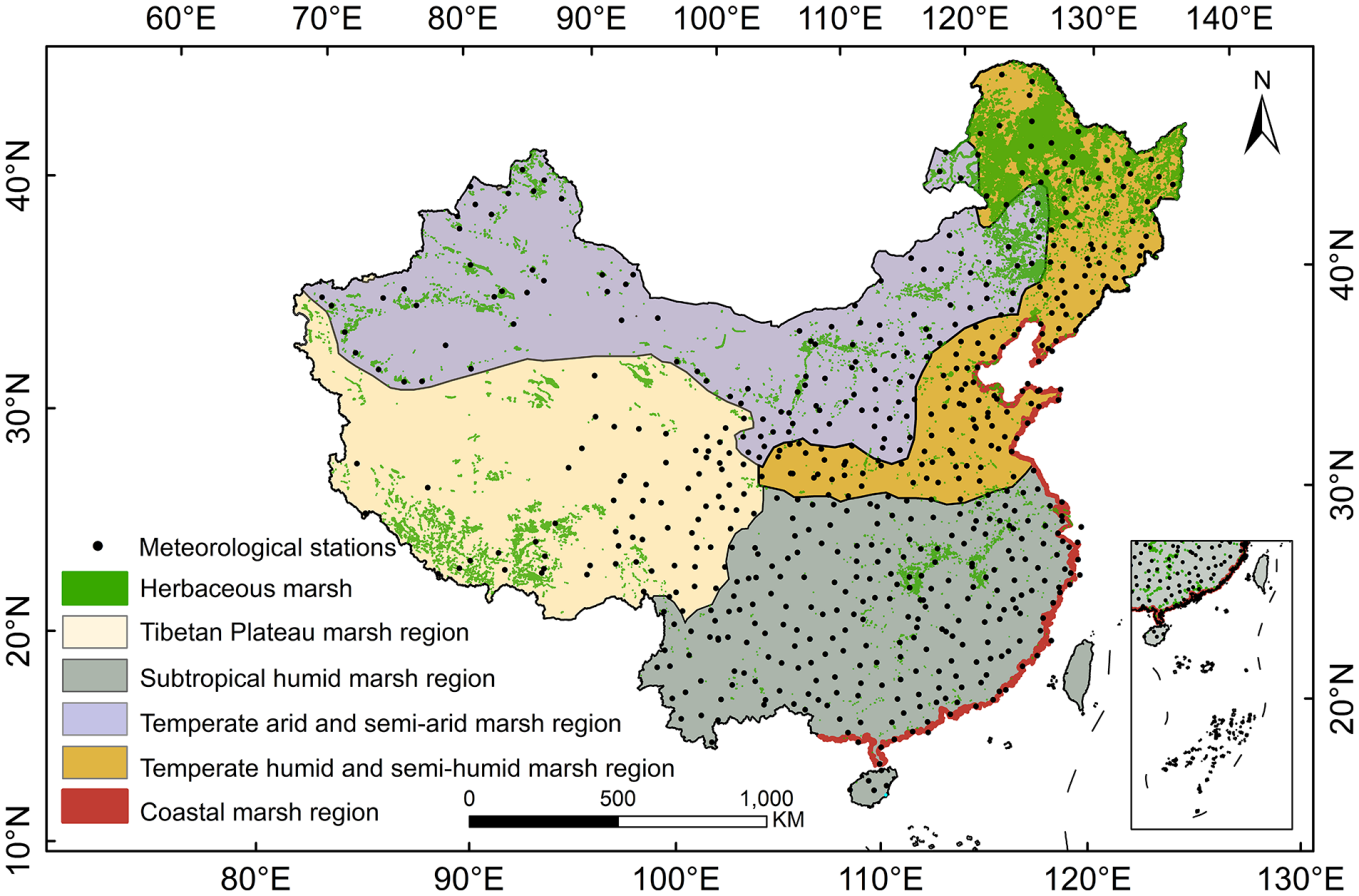
Distribution of herbaceous marsh in China and five marsh regions of China.

Herbaceous marshes are widely distributed in China. Their distribution can be divided into five sub-regions according to differences in geographical environment and topography: coastal (CST), temperate semi-humid and humid (THS), temperate semi-arid and arid (TAS), subtropical humid (SH), and Tibetan Plateau (TP) marsh regions (**Figure 1**) (Shen et al., 2021a).

The CST has a wide latitudinal range and is predominantly influenced by the East Asian monsoon. The northern region of the CST has lower precipitation and cooler temperatures than the southern region (Hao et al., 2020). Winter in the THS is characterized by low temperatures, rainfall and humidity, and summers are characterized by high temperatures, rainfall and humidity. The herbaceous marsh vegetation in the northern region of the THS mainly comprises *Carex* spp., *Deyeuxia angustifolia*, and *Phragmites australis*, and the dominant species are *Bolboschoenus yagara*, *Trapa incisa*, and *Nymphoides peltate* in the southern region of this region (Shen et al., 2021a). The TAS has high summer and low winter temperatures, with precipitation decreasing from east to west and being unevenly distributed seasonally (Hong et al., 2022b) and the dominant species of herbaceous marsh vegetation are *Elymus nutans*, *Suaeda glauca*, and *Phragmites australis* (Shen et al., 2021a). Temperatures are high in summer and mid-range in winter in the SH and rainfall is high in both seasons (Ren et al., 2021). Annual rainfall generally decreases from southeast to northwest, and the region has abundant light, heat, and water resources (Liu et al., 2021). The main species of herbaceous marsh vegetation in the SH are *Polygonum hydropiper*, *Miscanthus lutarioriparius* and *Zizania latifolia* (Shen et al., 2021a). The average altitude of the TP is above 4000 m, with higher altitudes in the northwest and lower altitudes in the southeast. Average annual precipitation gradually increases from northwest to southeast, and average annual temperature gradually decreases from southeast to northeast (Sun et al., 2022). The dominant species of herbaceous marsh vegetation in the TP are *Kobresia littledalei*, *Blysmus sinocompressus*, and *Phragmites australis* (Shen et al., 2021a).

### 2.2 Data

Annual NPP (MOD17A3) data from 2000 to 2020 were obtained from the National Aeronautics and Space Administration (https://ladsweb.modaps.eosdis.nasa.gov). Spatial resolution of the data is 500 m, and it has been tested for quality assurance (Shen et al., 2022b). The distribution of herbaceous marshes in China were obtained from the 2010-2015 dataset provided by the China Wetland Ecology and Environment Data Center (http://wdcrre.data.ac.cn/) , which have been verified by field observation (Liu et al., 2023a). Meteorological data used in this study were monthly average maximum temperature, average minimum temperature, average temperature, and precipitation data, which were obtained from the National Meteorological Center (http://data.cma.cn/en). To ensure the continuity of monthly climate data, this study finally selected and used meteorological data from 613 meteorological stations with no missing data at each station during the whole study period in China (**Figure 1**), and there are 23 meteorological stations located in the herbaceous marsh regions of China.

### 2.3 Methods

Monthly climate data (maximum temperature (T_max_), minimum temperature (T_min_), mean temperature (T_mean_), and precipitation) were spatially interpolated using the ordinary kriging method to obtain raster data, which were harmonized with the NPP data (Shen et al., 2022b). Seasonal meteorological data were calculated for spring (March, April, and May), summer (June, July, and August), autumn (September, October, and November), and winter (December, January, and February) using monthly meteorological data (Shen et al., 2021b). The regional mean value of each variable was calculated from the average of all the pixels in herbaceous marshes of this region (Shen et al., 2022a). Consistent with previous studies (Ma et al., 2023; Liu et al., 2023b; Shen et al., 2024), a linear regression analysis was used to calculate the trends of NPP and meteorological factors over time, using the following formula (**Equation 1**):


(1) $$\theta_{slope}=\frac{\left(t*\sum_{r=1}^{t}r*B_{r}\right)-\left(\sum_{r=1}^{t}r\sum_{r=1}^{t}B_{r}\right)}{t*\sum_{r=1}^{t}r^{2}-{\left(\sum_{r=1}^{t}r\right)}^{2}}$$

Where *θ*_slope_ is the trend of NPP or meteorological factor; *t* is the length of the time series of the study (21 year); *r* is the year number; ${\text{B}}_{\text{r}}$ is the NPP or meteorological factor for year *r*. If *θ*_slope_ is positive, it means that the change in the NPP or meteorological factor is positive, and vice versa, it is a negative trend. If *θ*_slope_ is 0, it indicates no change.

Consistent with a number of earlier research (Shen et al., 2016; Huang et al., 2020), we calculated partial correlations between NPP with meteorological factors in order to assess the impact of climatic change on NPP. This partial correlation method can effectively exclude the interference of other factors (Li and Qin, 2019; Ren et al., 2023; Shen et al., 2024), thus accurately reflecting the relationship between meteorological factors and NPP.

## 3 Results

### 3.1 Temporal and spatial changes in NPP of herbaceous marshes in China

**Figure 2:**
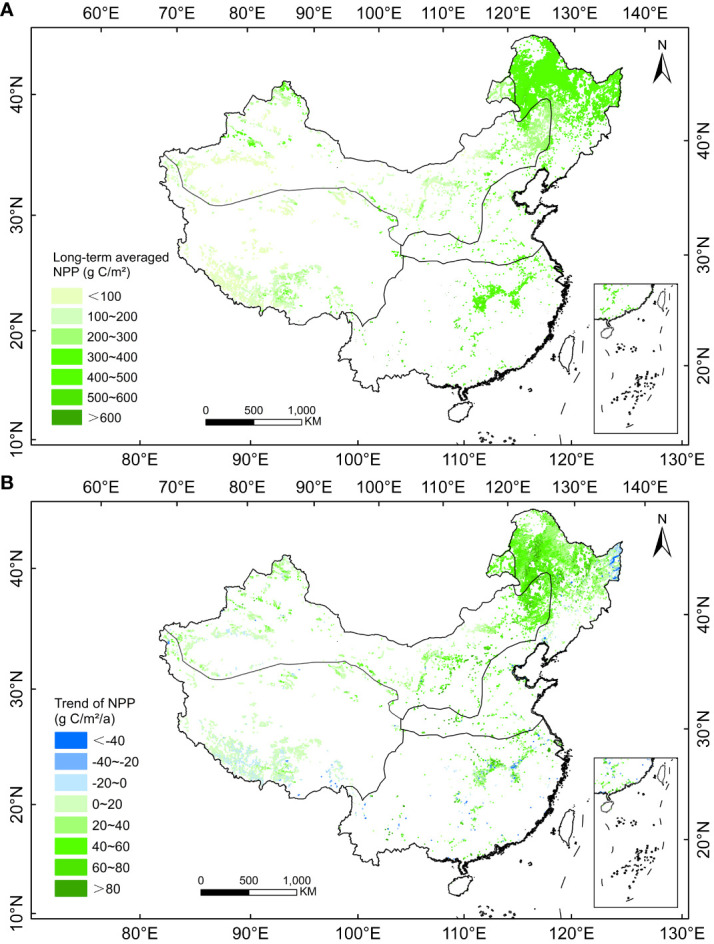
Spatial patterns in **(A)** long term average NPP and **(B)** temporal trend of the NPP of herbaceous marsh in China from 2000 to 2020.

There was spatial heterogeneity in the long-term average and trends in NPP of herbaceous marsh in various regions in China from 2000 to 2020 (**Figure 2**). The long-term average NPP of herbaceous marsh in China from 2000-2020 was 336.60 g C/m^2^ and was generally higher in the eastern region and lower in the western region of China (**Figure 2A**). Areas with high long-term average NPP were mainly located in the northern region of the THS and the central region of the SH (**Figure 2A**). Areas with low long-term average NPP were mainly located in the western region of the TAS and southwestern region of the TP (**Figure 2A**). Regional average NPP over the years 2000-2020 was 486.13, 402.00, 322.60, 238.94, and 141.01 g C/m^2^, in the SH, THS, CST, TAS, and TP marsh regions, respectively.

**Figure 3:**
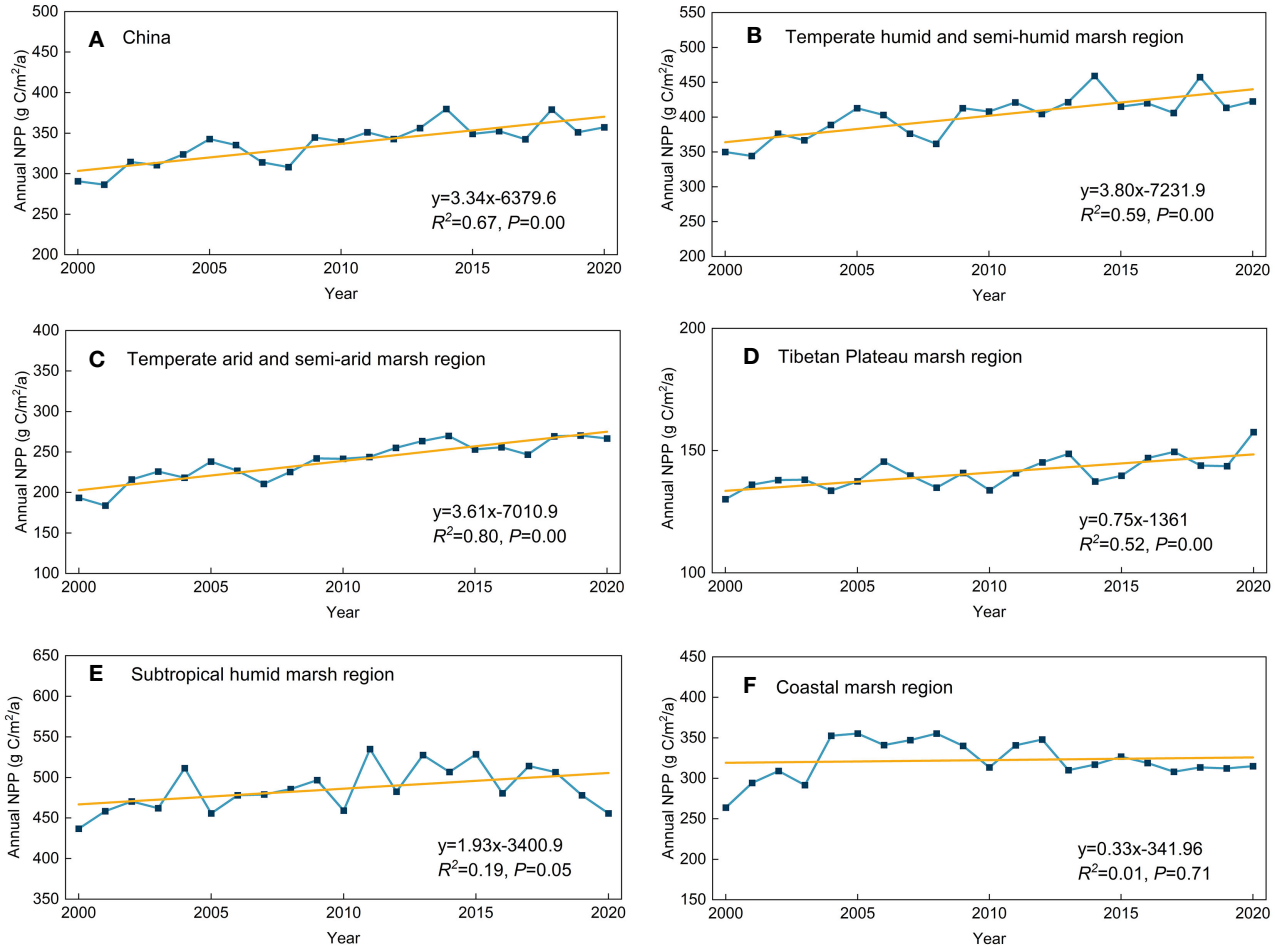
Temporal variations in regional average NPP of herbaceous marsh in **(A)** China and **(B–F)** different marsh regions of China from 2000-2020 (yellow line indicates the linear trend of NPP).

The regional long-term average NPP of herbaceous marshes in China increased significantly (*P<0.05*) by 3.34 g C/m^2^/a from 2000 to 2020, with significant (*P<0.05*) increase trends of 3.80, 3.61, 1.93, 0.75 g C/m^2^/a in the THS, TAS, SH, and TP, respectively (**Figure 3**). A weak increasing trend (0.33 g C/m^2^/a) of regional average NPP was found in the CST (**Figure 3**). Spatially, the upward trend in the NPP was most significant in the northern THS, eastern TAS, and central SH during the past two decades (**Figure 2B**). By contrast, a downward trend was observed for eastern THS and southern TP (**Figure 2B**).

### 3.2 Trends in meteorological factors

**Figure 4:**
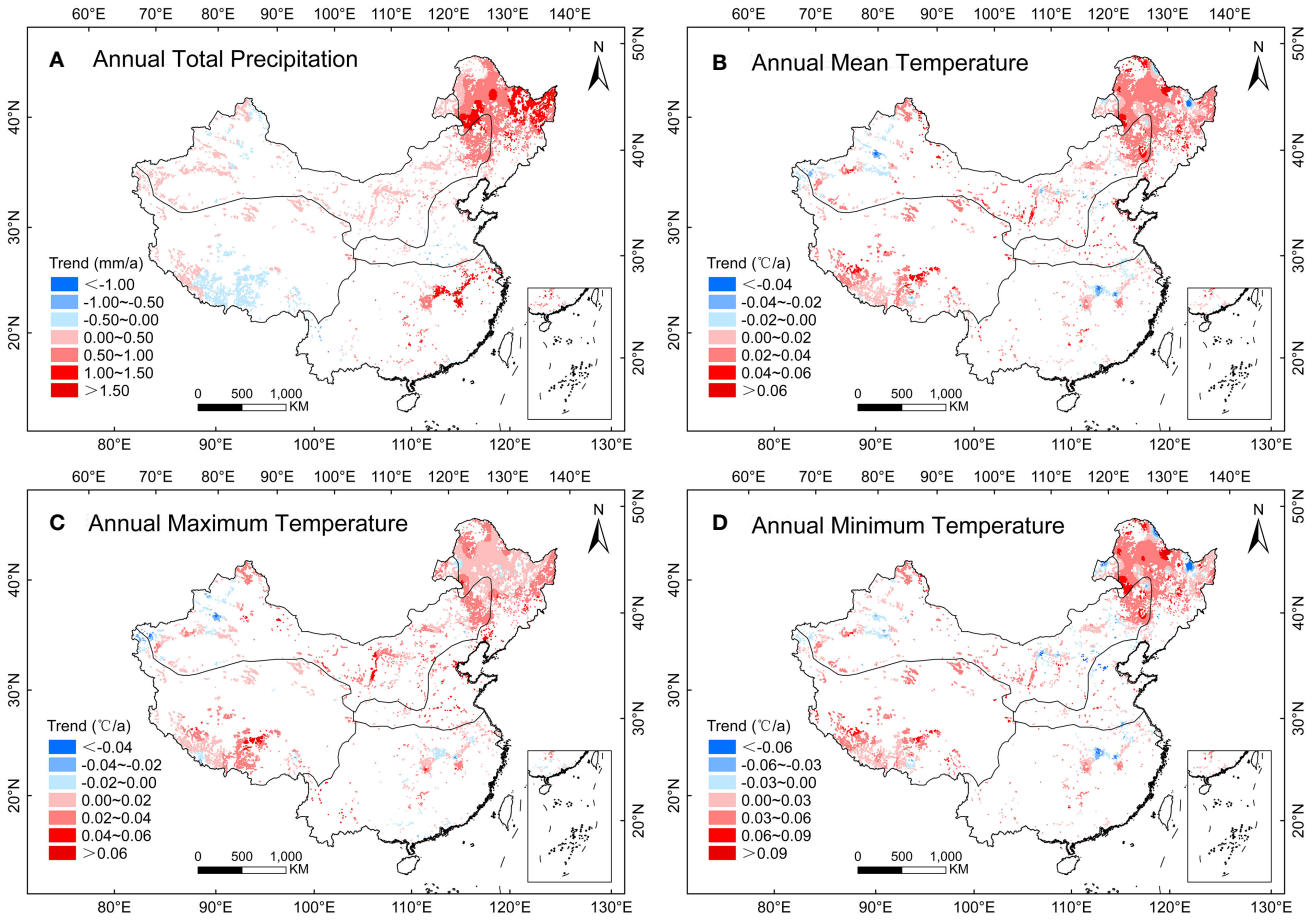
Variation trends of annual **(A)** precipitation (mm/a), **(B)** mean temperature (°C/a), **(C)** maximum temperature (°C/a), and **(D) ** minimum temperature(°C/a) in herbaceous marshes of China during 2000 - 2020.

There was a highly significant (*P<0.01*) increase in annual total precipitation across the herbaceous marsh regions of China from 2000 to 2020 (**Table 1**; **Figure 4**). At the regional level, the positive trend in annual precipitation was significant (*P<0.01*) in the THS (0.84 mm/a) and the TAS (0.39 mm/a) (**Table 1**). There was a significant positive trend in the TP for annual T_mean_ (*P<0.05*) and T_min_ (*P<0.01*). In different seasons, there were significant (*P<0.05*) positive trends in summer and autumn precipitation in all the herbaceous marsh distribution regions (**Table 1**). There were significant (*P<0.05*) positive trends of average spring T_min_ (0.05 mm/a), and T_mean_ and T_min_ in summer (0.03 mm/a, 0.05 mm/a) and in autumn (0.06 mm/a, 0.08 mm/a) in the TP, with the summer T_min_ and autumn T_min_ showing highly significant (*P<0.01*) positive trends (**Table 1**).

**Table 1:** Trends in annual and seasonal mean precipitation (mm/a) and temperatures (°C/a) in different herbaceous marsh regions of China from 2000-2020.

	China marsh region	Temperate humid and semi-humid marsh region	Temperate arid and semi-arid marsh region	Tibetan Plateau marsh region	Subtropical humid marsh region	Coastal marsh region
Annual total precipitation	0.62**	0.84**	0.39**	0.19	1.10	0.47
Annual mean temperature	0.02	0.03	0.02	0.03*	0.01	0.04*
Annual maximum temperature	0.02	0.02	0.02	0.02	0.02	0.04*
Annual minimum temperature	0.03*	0.04	0.02	0.05**	0.02	0.02
Spring precipitation	0.12	0.10	0.22	-0.05	0.55	0.29
Spring mean temperature	0.04	0.04	0.05	0.03	0.02	0.05
Spring maximum temperature	0.05	0.06	0.05	0.01	0.03	0.05
Spring minimum temperature	0.03	0.02	0.03	0.05*	0.02	0.03
Summer precipitation	1.57**	2.15*	0.87**	0.33	2.84*	1.78*
Summer mean temperature	-0.01	-0.02	-0.01	0.03*	0.00	0.03
Summer maximum temperature	-0.03	-0.05	-0.02	0.03	0.01	0.03
Summer minimum temperature	0.03*	0.03	0.02	0.05**	0.01	0.02
Autumn precipitation	0.82**	1.17**	0.49*	0.18	1.15	-0.22
Autumn mean temperature	0.02	0.02	0.00	0.06*	0.01	0.03
Autumn maximum temperature	0.00	-0.01	-0.01	0.04*	0.00	0.04
Autumn minimum temperature	0.04	0.05	0.01	0.08**	0.03	0.02
Winter precipitation	-0.04	-0.07	-0.02	0.10	-0.15	0.04
Winter mean temperature	0.04	0.06	0.02	0.02	0.02	0.04
Winter maximum temperature	0.04	0.05	0.03	-0.01	0.03	0.06
Winter minimum temperature	0.04	0.05	0.02	0.04	0.02	0.03

Significant at ** *P*<0.01 and * *P*<0.05 levels (the same below).

### 3.3 Correlation between meteorological factors and NPP

**Figure 5:**
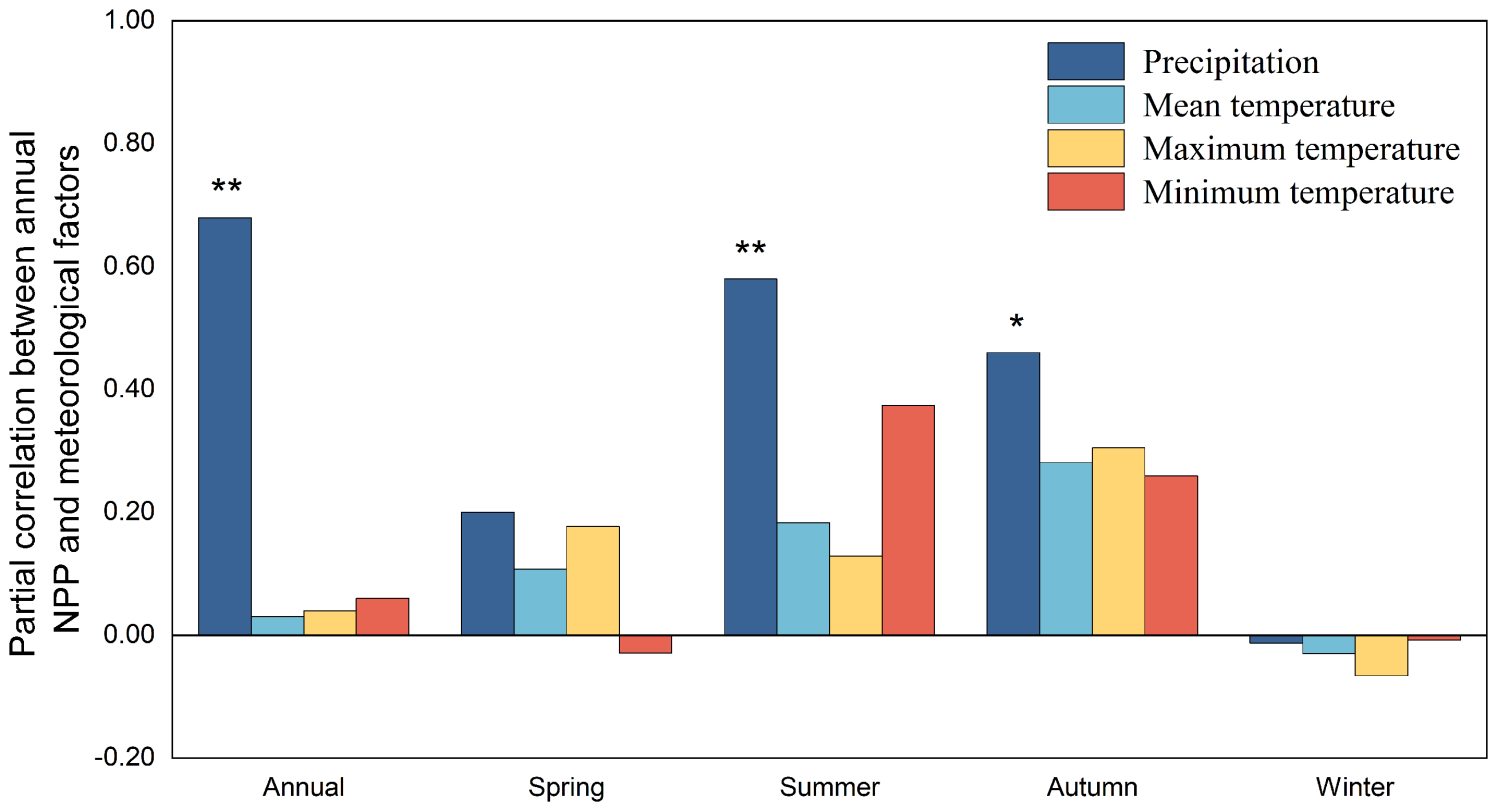
Partial correlations between the NPP of herbaceous marsh and meteorological factors in China from 2000 to 2020. Significant at ** P<0.01 and * P<0.05 levels.

**Figure 6:**
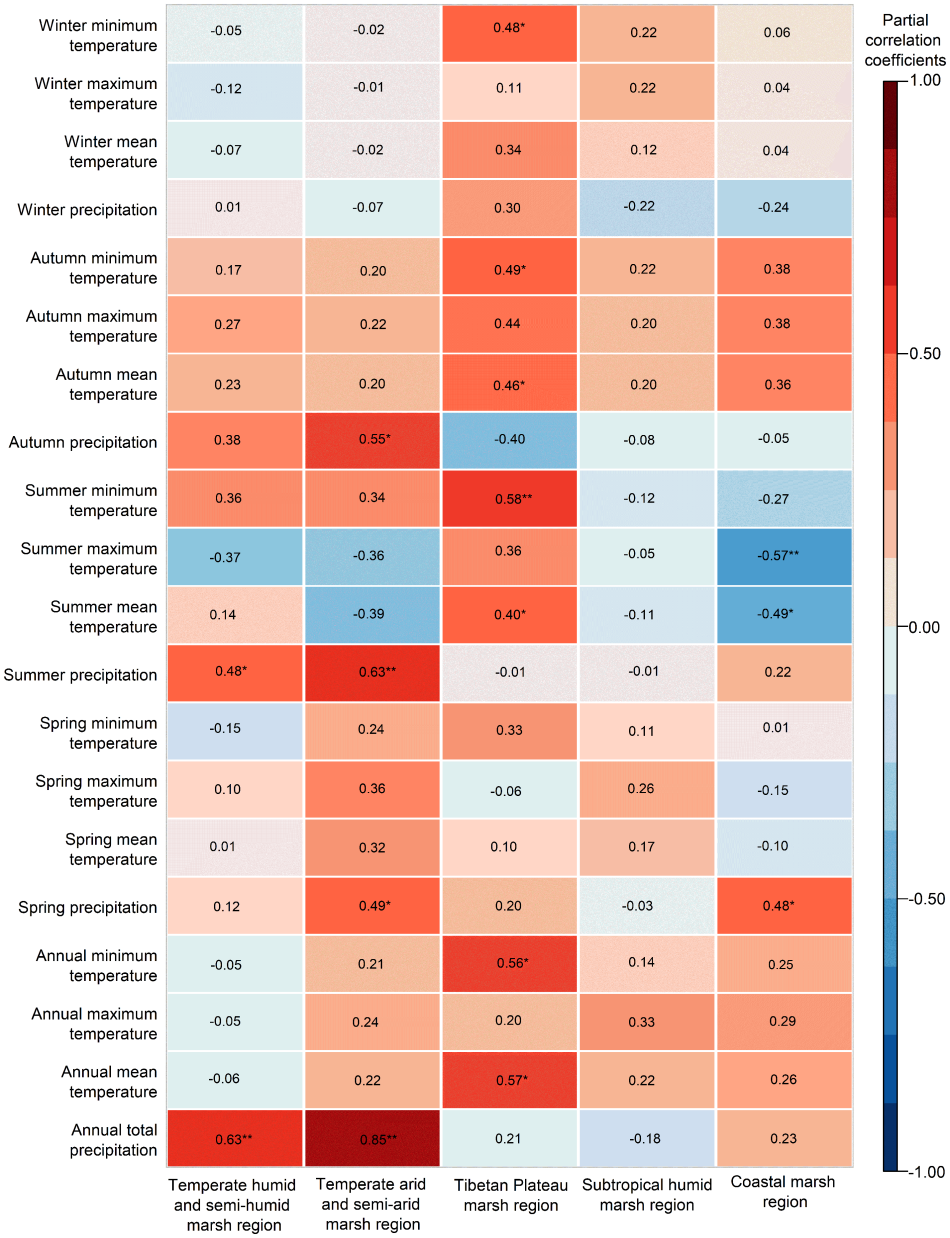
Partial correlations between the marsh NPP and meteorological factors in different herbaceous marsh regions of China from 2000 to 2020.

The NPP of herbaceous marshes in China was significantly (*P<0.05*) positive correlation with annual precipitation from 2000 to 2020, and the correlation was larger in the THS and TAS (**Figures 5**, **6**). The NPP of herbaceous marshes in the TP exhibited a positive correlation with annual T_min_ and T_mean_, with the latter correlation significant (**Figure 6**).

Across China, there was a significant (*P<0.05*) positive correlation between NPP with summer and autumn precipitation (**Figure 5**). At the regional level, NPP and summer precipitation was found to have significant (*P<0.01*) and positive correlation in the THS and TAS and it with spring and autumn precipitation was found to have significant (*P<0.05*) and positive correlation in the TAS (**Figure 6**).

The NPP of herbaceous marsh in China had a moderate negative correlation with summer T_mean_ and T_max_, and a moderate positive correlation with summer T_min_. At the regional level (**Figure 6**), the NPP of herbaceous marsh was moderately positively correlated with summer T_min_ in the THS, and moderately and significantly (*P<0.05*) negatively correlated with summer T_max_ in the THS and TAS, respectively. In the TP, the NPP correlated significantly (*P<0.05*) and positively with T_mean_ and T_min_ (**Figure 6**) and moderately positively with summer T_max_ (**Figure 6**). The NPP was also significantly (*P<0.01*) positively correlated with autumn T_mean_ and autumn and winter T_min_, and was most highly correlated with autumn T_min_ (**Figure 6**). In the CST, herbaceous marsh NPP was significantly and negatively correlated with all meteorological factors, with the correlation with summer T_max_ reaching the highly significant (*P<0.01*) level (**Figure 6**). The marsh NPP was not significantly correlated with temperature or precipitation in the SH (**Figure 6**).

**Figure 7:**
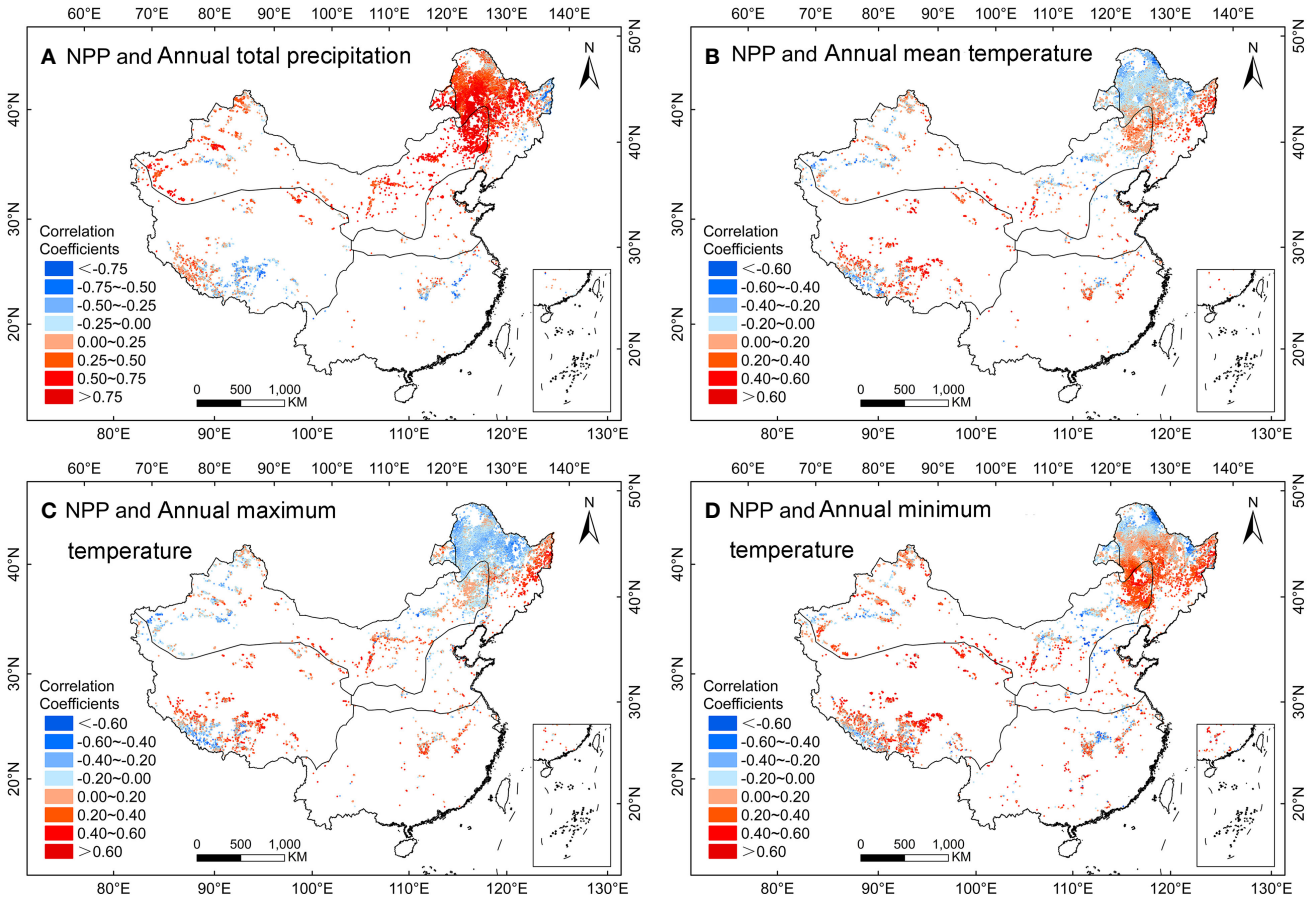
Spatial patterns in the partial correlation coefficients between NPP and annual **(A)** precipitation, **(B)** mean temperature, **(C)** maximum temperature, and **(D)** minimum temperature in herbaceous marshes of China during 2000 - 2020.

A significant (*P<0.05*) and positive correlation was found between NPP and annual precipitation over the whole herbaceous marsh in China from 2000 to 2020 (**Figure 7**). However, a negative correlation was found in the southern region of the TP (**Figure 7**). The NPP and annual T_mean_, T_max_, and T_min_ were observed a negative correlation in the northern region of THS (**Figure 7**).

Partial correlations between herbaceous marsh NPP and meteorological factors differed across seasons and regions. The NPP was negatively correlated with temperature in summer in most of the regions. However, NPP was positively correlated with summer T_mean_ and summer T_max_ in the northern region of the TP (**Supplementary Figure S6**). The NPP and winter temperatures (T_mean_, T_max_, and T_min_) were positively correlated across most of the regions, but were negatively correlated in the northern region of the THS (**Supplementary Figure S8**).

## 4 Discussion

### 4.1 Temporal and spatial variation of NPP

We determined that the long-term average NPP of herbaceous marsh in China was 336.60 g C/m^2^ between 2000 and 2020. This result was higher than the long-term average NPP of 282.00 g C/m^2^ for grasslands in China calculated by Zhou et al. (2020), but close to the long-term average NPP of 339.85 g C/m^2^ for marsh in Inner Mongolian of China calculated by Wang et al. (2023). The reason may be because that large areas of desert grasslands and alpine grasslands were included in the study by Zhou et al. (2020) and those vegetations are generally less productive than the wetter herbaceous marshes (Shen et al., 2021a). Furthermore, herbaceous marshes are wetter than grasslands and provide better moisture conditions for vegetation growth (Liu et al., 2023b). Vegetation grows more luxuriantly, making the herbaceous marsh NPP higher than the grassland NPP. The areas with higher long-term average herbaceous marsh NPP were mainly located in the northern region of the THS and the central region of the SH (**Figure 2A**), likely due to the beneficial hydrothermal conditions for herbaceous vegetation growth in these regions (Shen et al., 2021a). The regions with low long-term average herbaceous marsh NPP were mainly located in the relatively arid western region of the TAS (Huang et al., 2019), and the cool southwest region of the TP (Shen et al., 2022b) (**Figure 2A**). As a result, vegetation grows shorter in these regions (Shen et al., 2022b), and thus the long-term averaged vegetation NPP is lower in the western region of the TAS and the southwest region of the TP. Piao et al. (2020) analyzed leaf area index of vegetation in China over the last 20 years and found that overall growth conditions for vegetation in China have improved. In this study, we confirmed that the growth conditions for herbaceous marsh vegetation have improved significantly in China during the last two decades (**Figure 2B**).

### 4.2 Response of the NPP of herbaceous marsh to climatic factors

Our study found that the NPP of Chinese herbaceous marsh during 2000 to 2020 showed a strong and statistically significant (*P<0.01*) positive correlation with summer and autumn precipitation (**Figure 5**), indicating that ongoing increased precipitation in summer and autumn could lead to an increase in the national average NPP across China.

At the regional level, in the herbaceous marshes of THS, there was a significant (*P<0.05*) positive correlation between summer precipitation with NPP (**Figure 6**). It is likely attributable to the large areas of seasonal marsh in these regions (Poiani et al., 1995; Mitsch et al., 2010). Increased summer precipitation can lead to a rise in area of marsh, which in turn leads to an increase in marsh NPP at a certain extent (500 m × 500 m) (Niu et al., 2012). Consequently, this results in an increase in NPP in this region (Martina et al., 2016). On one hand, more precipitation could lead to more seasonal marsh distributions in these areas (Poiani et al., 1995; Mitsch et al., 2010), causing an increase in marsh NPP. On the other hand, an increase in summer precipitation in can also increase the water use efficiency of vegetation in the THS (Zheng et al., 2019), partly explaining the positive effects of summer precipitation on NPP in this region. In contrast, we found that an increase in summer T_max_ was associated with a significantly reduced NPP in the THS (**Figure 6**) likely due to increased evapotranspiration at the higher daytime temperatures (Shen et al., 2021b; Wang et al., 2022b). In addition, we found a differential effects of summer temperatures on the NPP of herbaceous marshes in the THS. Summer T_max_ in the THS was exhibited a moderate negative association with herbaceous marsh NPP, whereas summer T_min_ showed a moderate positive association with NPP in this region. It indicates that the increase in nighttime T_min_ increases the productivity of marsh vegetation. The increase in night T_min_ during the summer can promote respiration at night in marsh vegetation in the THS (Fares et al., 2011). However, increased T_min_ can also cause vegetation to produce more organic matter through a compensatory effect (Wang et al., 2023). The compensatory effect is a phenomenon that vegetation produces more organic matter the next day after consuming organic matter due to nighttime warming, resulting in some recovery of vegetation growth (Peng et al., 2013; Ulrich et al., 2019). Previous studies have shown that environments with sufficient water easily lead to a compensatory effect and even a super compensatory effect (Liu et al., 2023b; Shen et al., 2024), which can recover and even exceed the original state of the vegetation (Wang et al., 2023; Liu et al., 2023a). The subject of this study was marsh wetland with sufficient water and nutrients (Shen et al., 2022b); therefore, a super compensatory effect may have occurred in this region. This may explain the reason why the increase in T_min_ led to the increase in NPP. The NPP in the northern region of the THS was negatively correlated with winter temperatures (including T_mean_, T_max_, and T_min_) (**Supplementary Figure S8**), suggesting that an increase in winter temperatures is not conducive to an increase in NPP in this region. The warming of winter may have reduced the chilling of vegetation (Piao et al., 2011), which may have resulted in delayed growth and flowering. This may partly explain the reasons why winter warming can reduce NPP.

In the herbaceous marshes of TAS, the NPP was positively correlated with spring precipitation (**Figure 6**). The environment of TAS are more arid, and precipitation is the limiting factor for the growth of vegetation in this region (Wang et al., 2023). Increased spring precipitation can effectively alleviate the drought stress suffered by the vegetation, and is beneficial to the growth of the vegetation (Abel et al., 2023; Zhu et al., 2023; Shen et al., 2024). This could explain why the increase in spring precipitation leads to an increase in NPP in the temperate semi-arid and arid marsh regions. The NPP in the eastern and central regions of the TAS was positively correlated with spring temperatures (including T_mean_, T_max_, and T_min_) (**Supplementary Figure S5**). In these regions, warmer spring temperatures may reduce frost damage and promote heat accumulation in vegetation, thereby promoting vegetation growth (Hong et al., 2022a; Liu et al., 2023b).

In the herbaceous marshes of TP, the NPP was positively correlated with summer temperatures (T_mean_, T_max_, and T_min_) (**Figure 6**). Summer is the most favorable season for marsh vegetation growth (Bertness and Ellison, 1987; Forbrich et al., 2018), and higher daytime temperatures in summer promote photosynthesis by promoting enzyme activity (Peng et al., 2013; Smith and Dukes, 2013; Zandalinas et al., 2017; Daniel et al., 2020). Higher night time temperatures in summer can promote vegetation respiration at night (Wan et al., 2009). Wetlands are prone to an over compensatory effect (Maschinski and Whitham, 1989; Shen et al., 2022b), and the vegetation could accumulate more material by photosynthesis the following day than it consumes by respiration during the night (Wang et al., 2022a). This results in the accumulation of material and an increase in NPP. The NPP in the TP was significantly and positively correlated with autumn T_mean_ (**Figure 6**). An increase in autumn temperatures leads to a delay in the yellowing or senescence of vegetation leaves, resulting in a longer growing season (Shen et al., 2022a; Huang et al., 2023) and an increase in NPP. The NPP on the TP was generally positively correlated with T_min_ in autumn and winter (**Figure 6**), due to a reduction in freezing-induced damage to vegetation (Shen et al., 2022b). The NPP in the southern region of the TP was negatively correlated with annual total precipitation (**Figure 7**). This may be because of the higher altitude and lower temperatures in the area (Zhong et al., 2019), where increased precipitation can lead to lower temperatures (Ye et al., 2013) and the caused frost damage could result in a decrease in NPP (Wang et al., 2021).

In the herbaceous marshes of CST, NPP was positively correlated with spring precipitation (**Figure 6**), and was negatively correlated with summer T_mean_ and T_max_ in the coastal region. The increased spring precipitation may reduce the accumulation of salts at the surface and increase the activity of marsh vegetative root system in the CST (Suttle et al., 2007; Chu et al., 2019), thereby increasing the NPP of marsh vegetation in this region. Higher summer temperatures increase plant growth rates and biomass in the CST (Suttle et al., 2007; Chu et al., 2019). Additionally, warmer summer temperatures increase evapotranspiration and reduce soil moisture content (Wetherald and Manabe, 1995; Guan et al., 2011), resulting in increased salinity and leading to a decrease in plant biomass and growth rates in this region (Moffett et al., 2010; Tian et al., 2019).

In the herbaceous marshes of SH, the NPP was not significantly correlated with precipitation or temperature. This may be because the SH has beneficial hydrothermal conditions which do not limit the growth of marsh vegetation (Shen et al., 2021b).

### 4.3 Variation in vegetation of herbaceous marsh of China

From 2000 to 2020, there were increasing trends of annual and summer precipitation in the herbaceous marshes of China (**Table 1**). Based on the observed correlations between climatic factors and NPP in the herbaceous marsh areas of China, we can conclude that the increases in precipitation may be partly responsible for the nationwide increase in herbaceous marsh NPP. Annual total precipitation and summer precipitation showed increasing trends in the TAS and the THS, and the annual mean temperature and annual minimum temperature showed increasing trends in the TP (**Table 1**). The increase in annual total precipitation and summer precipitation may partly explain an increase in the NPP in the TAS and THS, and the increase in annual mean and minimum temperature may partly explain an increase in the NPP in the TP. Spatially, the most significant trend in increasing herbaceous marsh NPP was observed in the northern region of the THS and the eastern region of the TAS (**Figure 2B**). It is interesting that a highly significant increase in annual total precipitation was observed in both the THS and TAS (**Table 1**). Therefore, the increase in annual total precipitation may partially explain the increase in NPP in both regions. The decreasing trend in the annual total precipitation was mainly concentrated in the eastern region of the THS, where annual herbaceous marsh NPP was negatively correlated with annual total precipitation. This possibly explains the decrease in herbaceous marsh NPP in this region. Herbaceous marsh NPP in the TP showed an overall increasing trend and was significantly and positively correlated with summer temperatures (T_mean_, T_max_, and T_min_), autumn T_mean_, and autumn T_min_. There was a significant trend towards increasing temperatures in this region, therefore, the increase in annual T_min_ and summer and autumn temperatures (T_mean_ and T_min_) may explain the increase in NPP in the TP to some extent. Previous studies indicate that the Tibetan Plateau and the temperate semi-arid and arid marsh regions will become warmer and wetter in the future (Liu et al., 2017; Zhang et al., 2022). Therefore, the NPP of herbaceous marshes would continue to increase to some extent in the future, especially in the southwestern region of the Tibetan Plateau and the western region of the temperate semi-arid and arid marsh regions of China.

### 4.4 Limitations

This study may have some limitations. First, the NPP data has a relatively low resolution and possibly cannot reflect the actual productivity of marsh vegetation within a 500 m × 500 m area. At the same time, there may be uncertainty in the distribution of herbaceous marshes, and more data on marshes are required to validate our findings. Second, the meteorological stations are relatively few and unevenly distributed in the marsh regions, which may led to some uncertainties in the results. Third, this study only analyzed the changes in annual NPP of herbaceous marsh vegetation, as well as the impact of precipitation and temperature on annual NPP in China. Environmental factors other than temperature and precipitation, including solar radiation, relative humidity, and human activities, may also affect the NPP of herbaceous marsh vegetation. Moreover, we did not analyze the NPP for different vegetation types and the responses of seasonal NPP to climate change in this paper. In the future, we need to further explore the NPP for different vegetation types and the responses of seasonal NPP to more environmental factors changes.

## 5 Conclusions

From 2000 to 2020, the NPP of herbaceous marshes in China increased significantly with a rate of 3.34 g C/m^2^/a. Increased precipitation will cause an increase in the national average NPP in China to some extent. At a regional scale, increased annual precipitation significantly increased the NPP in temperate semi-arid and arid and temperate semi-humid and humid marsh regions. For the first time, we discovered asymmetric effects of daytime and nighttime temperatures on NPP in herbaceous marshes of China. In the Tibetan Plateau, increased autumn daytime temperature, as well as summer daytime and nighttime temperatures could increase the NPP of herbaceous marshes. In the temperate semi-humid and humid marsh region, we found a differential effects of increasing nighttime and daytime temperatures on NPP during the summer: increased summer daytime temperature decreases NPP while increased summer nighttime temperature increases NPP in this region. This study highlights the different effects of seasonal climatic changes on NPP of herbaceous marshes in different regions of China, and suggests that the differential effects of daytime and nighttime temperatures should be considering in simulating the NPP of herbaceous marshes in terrestrial ecosystem models, especially in the context of global asymmetric diurnal warming (faster warming trend during the night than during the day).

## Data availability statement

The raw data supporting the conclusions of this article will be made available by the authors, without undue reservation.

## Author contributions

LW: Methodology, Writing – original draft. XS: Data curation, Methodology, Writing – review & editing. JZ: Methodology, Visualization, Writing – review & editing. YL: Methodology, Supervision, Writing – review & editing. CD: Writing – review & editing. RM: Supervision, Visualization, Writing – review & editing. XL: Writing – review & editing. MJ: Writing – review & editing.

## References

[B1] Abel C. Abdi A. M. Tagesson T. Horion S. Fensholt R. (2023). Contrasting ecosystem vegetation response in global drylands under drying and wetting conditions. Global Change Biol. 29, 3954–3969. doi: 10.1111/gcb.16745 37103433

[B2] Bertness M. D. Ellison A. M. (1987). Determinants of pattern in a new-england salt-marsh community. Ecol. Monogr. 57, 129–147. doi: 10.2307/1942621

[B3] Bhowmik S. (2022). Ecological and economic importance of wetlands and their vulnerability: a review. Res. Anthology Ecosystem Conserv. Preserving Biodiversity, 11–27. doi: 10.4018/978-1-6684-5678-1.ch002

[B4] Chu X. J. Han G. X. Xing Q. H. Xia J. Y. Sun B. Y. Li X. G.. (2019). Changes in plant biomass induced by soil moisture variability drive interannual variation in the net ecosystem CO2 exchange over a reclaimed coastal wetland. Agric. For. Meteorology 264, 138–148. doi: 10.1016/j.agrformet.2018.09.013

[B5] Clarkson B. R. Ausseil A. G. E. Gerbeaux P. (2013). Wetland ecosystem services. Ecosystem Serv. New Zealand: conditions Trends 1, 192–202.

[B6] Daniel T. C. Cox I. M. D. Maclean A. S. Gardner K. J. G. (2020). Global variation in diurnal asymmetry in temperature, cloud cover, specific humidity and precipitation and its association with leaf area index. Global Change Biol. 26, 7099–7111. doi: 10.1111/gcb.15336 32998181

[B7] Erwin K. L. (2009). Wetlands and global climate change: the role of wetland restoration in a changing world. Wetlands Ecol. Manage. 17, 71–84. doi: 10.1007/s11273-008-9119-1

[B8] Fares S. Mahmood T. Liu S. R. Loreto F. Centritto M. (2011). Influence of growth temperature and measuring temperature on isoprene emission, diffusive limitations of photosynthesis and respiration in hybrid poplars. Atmospheric Environ. 45, 155–161. doi: 10.1016/j.atmosenv.2010.09.036

[B9] Forbrich I. Giblin A. E. Hopkinson C. S. (2018). Constraining marsh carbon budgets using long-term C burial and contemporary atmospheric CO2 fluxes. J. Geophysical Research-Biogeosciences 123, 867–878. doi: 10.1002/2017JG004336

[B10] Gang C. C. Zhang Y. Z. Wang Z. Q. Chen Y. Z. Yang Y. Li J. L.. (2017). Modeling the dynamics of distribution, extent, and NPP of global terrestrial ecosystems in response to future climate change. Global Planetary Change 148, 153–165. doi: 10.1016/j.gloplacha.2016.12.007

[B11] Guan B. Yu J. Lu Z. Zhang Y. Wang X. H. (2011). Effects of water-salt stresses on seedling growth and activities of antioxidative enzyme of suaeda salsa in coastal wetlands of the yellow river delta. Huanjing Kexue 32, 2422–2429. 22619973

[B12] Hammer D. A. Bastian R. K. (2020). Wetlands ecosystems: natural water purifiers? Constructed Wetlands Wastewater Treat, 5–19. doi: 10.1201/9781003069850-3

[B13] Hao J. Xu G. Y. Luo L. Zhang,. Z. Yang,. H. L. Li H. Y. (2020). Quantifying the relative contribution of natural and human factors to vegetation coverage variation in coastal wetlands in China. Catena 188. doi: 10.1016/j.catena.2019.104429

[B14] Hirota M. Kawada K. Hu Q. W. Kato T. Tang Y. H. Mo W. H.. (2007). Net primary productivity and spatial distribution of vegetation in an alpine wetland, Qinghai-Tibetan Plateau. Limnology 8, 161–170. doi: 10.1007/s10201-007-0205-5

[B15] Hong S. Zhang Y. Yao Y. Meng F. Zhao Q. Zhang Y. (2022a). Contrasting temperature effects on the velocity of early-versus late-stage vegetation green-up in the Northern Hemisphere. Global Change Biol. 28, 6961–6972. doi: 10.1111/gcb.16414 36054628

[B16] Hong X. C. Huang F. Zhang H. W. Wang P. (2022b). Characterizing the turning points in ecosystem functioning and their linkages to drought and human activities over the arid and semi-arid regions of northern China. Remote Sens. 14, 5396. doi: 10.3390/rs14215396

[B17] Huang J. P. Ma J. R. Guan X. D. Li Y. He Y. L. (2019). Progress in semi-arid climate change studies in China. Adv. Atmospheric Sci. 36, 922–937. doi: 10.1007/s00376-018-8200-9

[B18] Huang Y. Jiang N. Shen M. G. Guo L. (2020). Effect of preseason diurnal temperature range on the start of vegetation growing season in the Northern Hemisphere. Ecol. Indic. 112, 106161. doi: 10.1016/j.ecolind.2020.106161

[B19] Huang Z. Zhou L. Chi Y. (2023). Spring phenology rather than climate dominates the trends in peak of growing season in the Northern Hemisphere. Global Change Biol. 29, 4543–4555. doi: 10.1111/gcb.16758 37198735

[B20] Jimenez K. Starr G. Staudhammer C. Schedlbauer J. Loescher H. Malone S.. (2012). Carbon dioxide exchange rates from short-and long-hydroperiod Everglades freshwater marsh. J. Geophysical Research: Biogeosciences 117. doi: 10.1029/2012JG002117

[B21] Li J. L. (2014). Assessing the spatiotemporal variation in distribution, extent and NPP of terrestrial ecosystems in response to climate change from 1911 to 2000. PloS One 8, e80394. doi: 10.1371/journal.pone.0080394 PMC3840029 24282539

[B22] Li Y. Qin Y. (2019). The response of net primary production to climate change: A case study in the 400 mm annual precipitation fluctuation zone in China. Int. J. Environ. Res. Public Health 16, 1497. doi: 10.3390/ijerph16091497 31035620 PMC6539075

[B23] Liu H. Li X. J. Mao F. J. Zhang M. Zhu D. E. He S. B.. (2021). Spatiotemporal evolution of fractional vegetation cover and its response to climate change based on MODIS data in the subtropical region of China. Remote Sens. 13, 913. doi: 10.3390/rs13050913

[B24] Liu J. Du H. B. Wu Z. F. He H. S. Wang L. Zong S. W. (2017). Recent and future changes in the combination of annual temperature and precipitation throughout China. Int. J. Climatology 37, 821–833. doi: 10.1002/joc.4742

[B25] Liu Y. Shen X. J. Zhang J. Q. Wang Y. J. Wu L. Y. Ma R.. (2023a). Spatiotemporal variation in vegetation phenology and its response to climate change in marshes of Sanjiang Plain, China. Ecol. Evol. 13, e9755. doi: 10.1002/ece3.9755 36699565 PMC9848817

[B26] Liu Y. Shen X. J. Zhang J. Q. Wang Y. J. Wu L. Y. Ma R.. (2023b). Variation in vegetation phenology and its response to climate change in marshes of inner Mongolian. Plants-Basel 12, 2072. doi: 10.3390/plants12112072 37299051 PMC10255120

[B27] Liu Y. Wu C. Wang X. Zhang Y. (2023c). Contrasting responses of peak vegetation growth to asymmetric warming: Evidences from FLUXNET and satellite observations. Global Change Biol. 29, 2363–2379. doi: 10.1111/gcb.16592 36695551

[B28] Ma M. Wang Q. Liu R. Zhao Y. Zhang D. (2023). Effects of climate change and human activities on vegetation coverage change in northern China considering extreme climate and time-lag and-accumulation effects. Sci. Total Environ. 860, 160527. doi: 10.1016/j.scitotenv.2022.160527 36460108

[B29] Martina J. P. Currie W. S. Goldberg D. E. Elgersma K. J. (2016). Nitrogen loading leads to increased carbon accretion in both invaded and uninvaded coastal wetlands. Ecosphere 7, e01459. doi: 10.1002/ecs2.1459

[B30] Maschinski J. Whitham T. G. (1989). The continuum of plant responses to herbivory: the influence of plant association, nuturient availability, and timing. Am. Nat. 134, 1–19. doi: 10.1086/284962

[B31] Mitra S. Wassmann R. Vlek P. L. (2005). An appraisal of global wetland area and its organic carbon stock. Curr. Sci. 88, 25–35.

[B32] Mitsch W. J. Nahlik A. Wolski P. Bernal B. Zhang L. Ramberg L. (2010). Tropical wetlands: seasonal hydrologic pulsing, carbon sequestration, and methane emissions. Wetlands Ecol. Manage. 18, 573–586. doi: 10.1007/s11273-009-9164-4

[B33] Moffett K. B. Wolf A. Berry J. A. Gorelick S. M. (2010). Salt marsh–atmosphere exchange of energy, water vapor, and carbon dioxide: Effects of tidal flooding and biophysical controls. Water Resour. Res 46, 10. doi: 10.1029/2009WR009041

[B34] Nayak R. K. Patel N. Dadhwal V. (2010). Estimation and analysis of terrestrial net primary productivity over India by remote-sensing-driven terrestrial biosphere model. Environ. Monit. Assess. 170, 195–213. doi: 10.1007/s10661-009-1226-9 19908154

[B35] Niu Z. G. Zhang H. Y. Wang X. W. Yao W. B. Zhou D. M. Zhao K. Y.. (2012). Mapping wetland changes in China between 1978 and 2008. Chin. Sci. Bull. 57, 2813–2823. doi: 10.1007/s11434-012-5093-3

[B36] Peng S. S. Piao S. L. Ciais P. Myneni R. B. Chen A. Chevallier F.. (2013). Asymmetric effects of daytime and night-time warming on Northern Hemisphere vegetation. Nature 501, 88–92. doi: 10.1038/nature12434 24005415

[B37] Piao S. L. Wang X. H. Ciais P. Zhu B. Wang T. Liu J. (2011). Changes in satellite-derived vegetation growth trend in temperate and boreal Eurasia from 1982 to 2006. Global Change Biol. 17, 3228–3239. doi: 10.1111/j.1365-2486.2011.02419.x

[B38] Piao S. L. Wang X. H. Park T. Chen C. Lian X. He Y.. (2020). Characteristics, drivers and feedbacks of global greening. Nat. Rev. Earth Environ. 1, 14–27. doi: 10.1038/s43017-019-0001-x

[B39] Poiani K. A. Johnson W. C. Kittel T. G. F. (1995). Sensitivity of a prairie wetland to increased temperature and seasonal precipitation changes. Water Resour. Bull. 31, 283–294. doi: 10.1111/j.1752-1688.1995.tb03380.x

[B40] Ren P. X. Liu Z. L. Zhou X. L. Peng C. H. Xiao J. F. Wang S. H.. (2021). Strong controls of daily minimum temperature on the autumn photosynthetic phenology of subtropical vegetation in China. For. Ecosyst. 8, 1–12. doi: 10.1186/s40663-021-00309-9 PMC8550766 34721934

[B41] Ren H. Wen Z. Liu Y. Lin Z. Han P. Shi H.. (2023). Vegetation response to changes in climate across different climate zones in China. Ecol. Indic. 155, 110932. doi: 10.1016/j.ecolind.2023.110932

[B42] Reyer C. Lasch-Born P. Suckow F. Gutsch M. Murawski A. Pilz T. (2014). Projections of regional changes in forest net primary productivity for different tree species in Europe driven by climate change and carbon dioxide. Ann. For. Sci. 71, 211–225. doi: 10.1007/s13595-013-0306-8

[B43] Salimi S. Almuktar S. Scholz M. (2021). Impact of climate change on wetland ecosystems: A critical review of experimental wetlands. J. Environ. Manage. 286. doi: 10.1016/j.jenvman.2021.112160 33611067

[B44] Shen X. J. Jiang M. Lu X. G. Liu X. T. Liu B. Zhang J. Q.. (2021a). Aboveground biomass and its spatial distribution pattern of herbaceous marsh vegetation in China. Sci. China Earth Sci. 64, 1115–1125. doi: 10.1007/s11430-020-9778-7

[B45] Shen X. J. Liu B. H. Jiang M. Lu X. G. (2020). Marshland loss warms local land surface temperature in China. Geophysical Res. Lett. 47, e2020GL087648. doi: 10.1029/2020GL087648

[B46] Shen X. J. Liu B. H. Jiang M. Wang Y. G. Wang L. Zhang J. Q.. (2021b). Spatiotemporal change of marsh vegetation and its response to climate change in China from 2000 to 2019. J. Geophysical Research: Biogeosciences 126, e2020JG006154. doi: 10.1029/2020JG006154

[B47] Shen X. J. Liu B. H. Li G. D. Wu Z. F. Jin Y. H. Yu P. J.. (2014). Spatiotemporal change of diurnal temperature range and its relationship with sunshine duration and precipitation in China. J. Geophysical Research: Atmospheres 119, 13–163. doi: 10.1002/2014JD022326

[B48] Shen X. J. Liu Y. W. Zhang J. Q. Wang Y. G. Ma R. Liu B. H.. (2022b). Asymmetric impacts of diurnal warming on vegetation carbon sequestration of marshes in the Qinghai Tibet Plateau. Global Biogeochemical Cycles 36, e2022GB007396. doi: 10.1029/2022GB007396

[B49] Shen M. G. Piao S. L. Chen X. Q. An S. Fu Y. S. Wang S. P.. (2016). Strong impacts of daily minimum temperature on the green-up date and summer greenness of the Tibetan Plateau. Global Change Biol. 22, 3057–3066. doi: 10.1111/gcb.13301 27103613

[B50] Shen X. J. Shen M. G. Wu C. Y. Peñuelas J. Ciais P. Zhang J. Q.. (2024). Critical role of water conditions in the responses of autumn phenology of marsh wetlands to climate change on the Tibetan Plateau. Global Change Biol. 30, e17097. doi: 10.1111/gcb.17097 38273510

[B51] Shen M. G. Wang S. P. Jiang N. Sun J. P. Cao R. Y. Ling X. F.. (2022a). Plant phenology changes and drivers on the Qinghai–Tibetan Plateau. Nat. Rev. Earth Environ. 3, 633–651. doi: 10.1038/s43017-022-00317-5

[B52] Shukla P. R. Skea J. Calvo Buendia E. Masson-Delmotte V. Pörtner H. O. Roberts D.. (2019). Climate Change and Land: an IPCC special report on climate change, desertification, land degradation, sustainable land management, food security, and greenhouse gas fluxes in terrestrial ecosystems. IPCC., 37–74.

[B53] Smith N. G. Dukes J. S. (2013). Plant respiration and photosynthesis in global-scale models: incorporating acclimation to temperature and CO2. Global Change Biol. 19, 45–63. doi: 10.1111/j.1365-2486.2012.02797.x 23504720

[B54] Sun H. Z. Chen Y. B. Xiong J. N. Ye C. H. Yong Z. W. Wang Y.. (2022). Relationships between climate change, phenology, edaphic factors, and net primary productivity across the Tibetan Plateau. Int. J. Appl. Earth Observation Geoinformation 107, 102708. doi: 10.1016/j.jag.2022.102708

[B55] Suttle K. B. Thomsen M. A. Power M. E. (2007). Species interactions reverse grassland responses to changing climate. Science 315, 640–642. doi: 10.1126/science.1136401 17272720

[B56] Tian X. Y. Chen M. Lu F. Wang A. D. Han G. X. Guan B. (2019). Response of growth and root biomass of Phragmites australis to water level and salt stress at different growth stages in the Yellow River Delta. Shengtaixue Zazhi 38, 404–411. doi: 10.13292/j.1000-4890.201902.034

[B57] Ulrich W. Hulisz P. Mantilla-Contreras J. Elvisto T. Piernik A. (2019). Compensatory effects stabilize the functioning of Baltic brackish and salt marsh plant communities. Estuarine Coast. Shelf Sci. 231, 106480. doi: 10.1016/j.ecss.2019.106480

[B58] Wan S. Q. Xia J. Y. Liu W. X. Niu S. L. (2009). Photosynthetic overcompensation under nocturnal warming enhances grassland carbon sequestration. Ecology 90, 2700–2710. doi: 10.1890/08-2026.1 19886480

[B59] Wang Y. G. Shen X. J. Jiang M. Lu X. G. (2020). Vegetation change and its response to climate change between 2000 and 2016 in marshes of the songnen plain, northeast China. Sustainability 12, 3569. doi: 10.3390/su12093569

[B60] Wang Y. J. Shen X. J. Jiang M. Tong S. Z. Lu X. G. (2021). Spatiotemporal change of aboveground biomass and its response to climate change in marshes of the Tibetan Plateau. Int. J. Appl. Earth Observation Geoinformation 102, 102385. doi: 10.1016/j.jag.2021.102385

[B61] Wang Y. G. Shen X. J. Jiang M. Tong S. Z. Lu X. G. (2022a). Daytime and nighttime temperatures exert different effects on vegetation net primary productivity of marshes in the western Songnen Plain. Ecol. Indic. 137, 108789. doi: 10.1016/j.ecolind.2022.108789

[B62] Wang Y. G. Shen X. J. Tong S. Z. Zhang M. Y. Jiang M. Lu X. G. (2022b). Aboveground biomass of wetland vegetation under climate change in the western songnen plain. Front. Plant Sci. 13. doi: 10.3389/fpls.2022.941689 PMC9247621 35783931

[B63] Wang Y. Zhang J. Shen X. Ma R. Liu Y. Wu L.. (2023). Spatiotemporal variation of marsh vegetation productivity and climatic effects in Inner Mongolia, China. Front. Ecol. Evol. 11. doi: 10.3389/fevo.2023.1138965

[B64] Wetherald R. T. Manabe S. (1995). The mechanisms of summer dryness induced by greenhouse warming. J. Climate 8, 3096–3108. doi: 10.1175/1520-0442(1995)008<3096:TMOSDI>2.0.CO;2

[B65] Woltz V. L. Stagg C. L. Byrd K. B. Windham-Myers L. Rovai A. S. Zhu Z. (2023). Above-and belowground biomass carbon stock and net primary productivity maps for tidal herbaceous marshes of the United States. Remote Sens. 15, 1697. doi: 10.3390/rs15061697

[B66] Ye S. Y. Pei L. X. He L. Xie L. J. Zhao G. M. Yuan H. M.. (2022). Wetlands in China: Evolution, carbon sequestrations and services, threats, and preservation/restoration. Water 14, 1152. doi: 10.3390/w14071152

[B67] Ye J. S. Reynolds J. F. Sun G. J. Li F. M. (2013). Impacts of increased variability in precipitation and air temperature on net primary productivity of the Tibetan Plateau: a modeling analysis. Climatic Change 119, 321–332. doi: 10.1007/s10584-013-0719-2

[B68] Yu J. B. Liu J. S. Meixner F. X. Wang J. D. Gao Y. J. Wang Y.. (2010). Estimating net primary productivity and nutrient stock in plant in freshwater marsh, northeastern China. Clean-Soil Air Water 38, 1080–1086. doi: 10.1002/clen.201000294

[B69] Zandalinas S. I. Mittler R. Balfagón D. Arbona V. Gómez-Cadenas A. (2017). Plant adaptations to the combination of drought and high temperatures. Physiologia Plantarum 162, 2–12. doi: 10.1111/ppl.12540 28042678

[B70] Zhang C. J. Ren Y. Y. Cao L. J. Wu J. Zhang S. Q. Hu C. Y.. (2022). Characteristics of dry-wet climate change in China during the past 60 years and its trends projection. Atmosphere 13, 275. doi: 10.3390/atmos13020275

[B71] Zheng H. Lin H. Zhu X. J. Jin Z. Bao H. (2019). Divergent spatial responses of plant and ecosystem water-use efficiency to climate and vegetation gradients in the Chinese Loess Plateau. Global Planetary Change 181, 102995. doi: 10.1016/j.gloplacha.2019.102995

[B72] Zhong L. Ma Y. M. Xue Y. K. Piao S. L. (2019). Climate change trends and impacts on vegetation greening over the tibetan plateau. J. Geophysical Research-Atmospheres 124, 7540–7552. doi: 10.1029/2019JD030481

[B73] Zhou D. M. Gong H. L. Wang Y. Y. Khan S. B. Zhao K. Y. (2009). Driving forces for the marsh wetland degradation in the honghe national nature reserve in sanjiang plain, northeast China. Environ. Modeling Assess. 14, 101–111. doi: 10.1007/s10666-007-9135-1

[B74] Zhou W. Li J. L. Yue T. X. Zhou W. Li J. Yue T. (2020). Spatial–Temporal Dynamics of Grassland Net Primary Productivity in china and Its Response to Climate Factors. Remote Sensing Monitoring and Evaluation of Degraded Grassland in CHINA: Accounting of Grassland Carbon Source and Carbon Sink. Springer-Verlag Singapore Pte Ltd, Singapore, 39–54. doi: 10.1007/978-981-32-9382-3_3

[B75] Zhu Z. Wang H. Harrison S. P. Prentice I. C. Qiao S. Tan S. (2023). Optimality principles explaining divergent responses of alpine vegetation to environmental change. Global Change Biol. 29, 126–142. doi: 10.1111/gcb.16459 PMC10092415 36176241

