# Supplementary material for: Spatial and temporal variation of net primary productivity of herbaceous marshes and its climatic drivers in China
Source: Front Plant Sci. 2024 May 14;15:1380081. doi: 10.3389/fpls.2024.1380081 (PMC11130473; doi:10.3389/fpls.2024.1380081)
Supplement: Supplementary file 1 [file DataSheet_1.docx]

**Supporting Information for** **"Spatial and temporal variation of net primary productivity of herbaceous marshes and its climatic drivers in China"**

**Contents of this file**

**Figure S1.** Variation trends of spring precipitation (mm/a), T_mean_ (°C/a), T_max_ (°C/a) and T_min_ (°C/a) in herbaceous marshes of China during 2000 - 2020.

**Figure S2.** Variation trends of summer precipitation (mm/a), T_mean_ (°C/a), T_max_ (°C/a) and T_min_ (°C/a) in herbaceous marshes of China during 2000 - 2020.

**Figure S3.** Variation trends of autumn precipitation (mm/a), T_mean_ (°C/a), T_max_ (°C/a) and T_min_ (°C/a) in herbaceous marshes of China during 2000 - 2020.

**Figure S4.** Variation trends of winter precipitation (mm/a), T_mean_ (°C/a), T_max_ (°C/a) and T_min_ (°C/a) in herbaceous marshes of China during 2000 - 2020.

**Figure S5.** Spatial patterns in the correlation coefficients between NPP and spring climatic factors in herbaceous marshes of China during 2000 - 2020.

**Figure S6.** Spatial patterns in the correlation coefficients between NPP and summer climatic factors in herbaceous marshes of China during 2000 - 2020.

**Figure S7.** Spatial patterns in the correlation coefficients between NPP and autumn climatic factors in herbaceous marshes of China during 2000 - 2020.

**Figure S8.** Spatial patterns in the correlation coefficients between NPP and winter climatic factors in herbaceous marshes of China during 2000 - 2020.


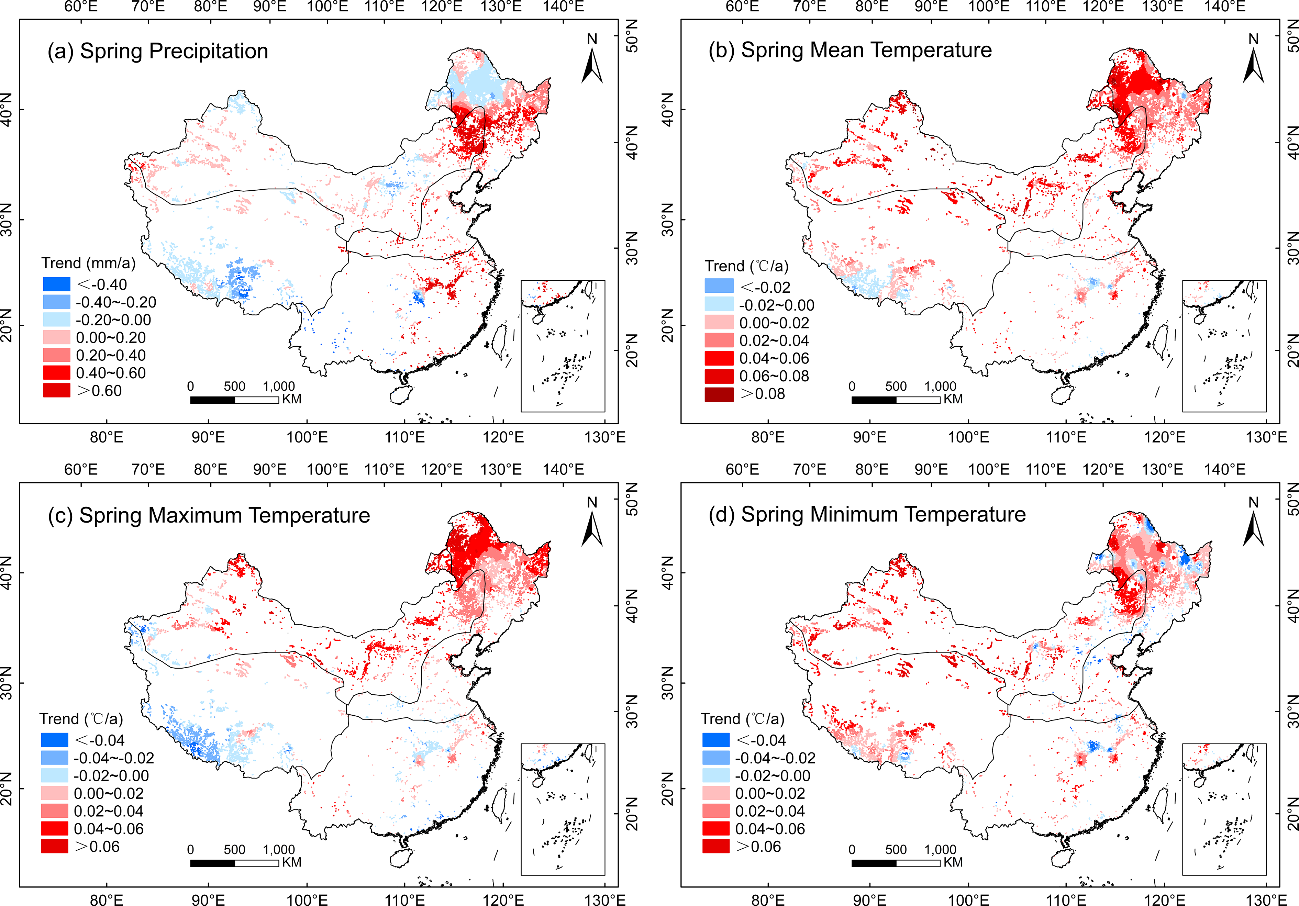


**Figure S1.** Variation trends of spring precipitation (mm/a), T_mean_ (°C/a), T_max_ (°C/a), and T_min_ (°C/a) in herbaceous marshes of China during 2000 - 2020.


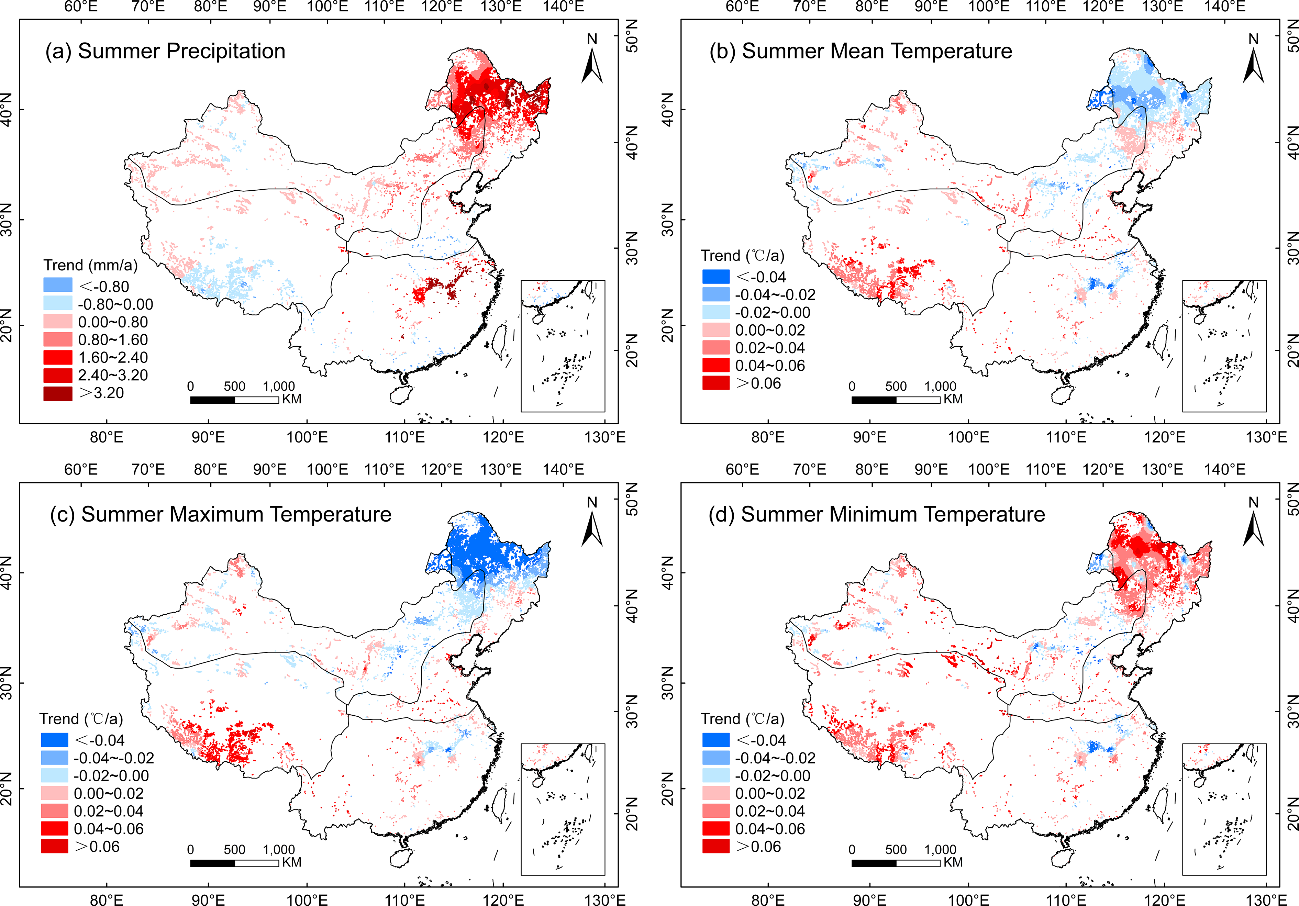


**Figure S2.** Variation trends of summer precipitation (mm/a), T_mean_ (°C/a), T_max_ (°C/a), and T_min_ (°C/a) in herbaceous marshes of China during 2000 - 2020.


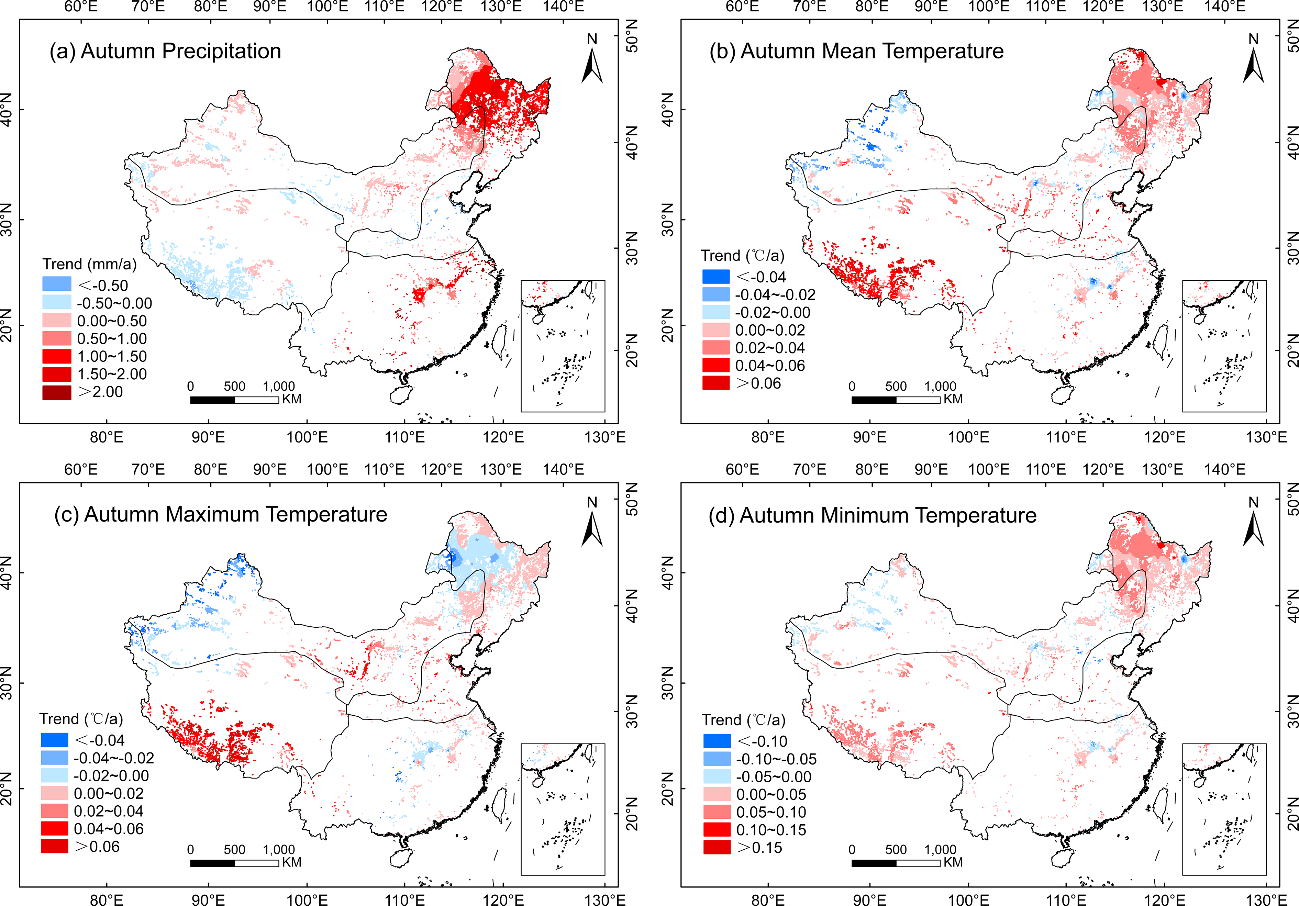


**Figure S3.** Variation trends of autumn precipitation (mm/a), T_mean_ (°C/a), T_max_ (°C/a), and T_min_ (°C/a) in herbaceous marshes of China during 2000 - 2020.


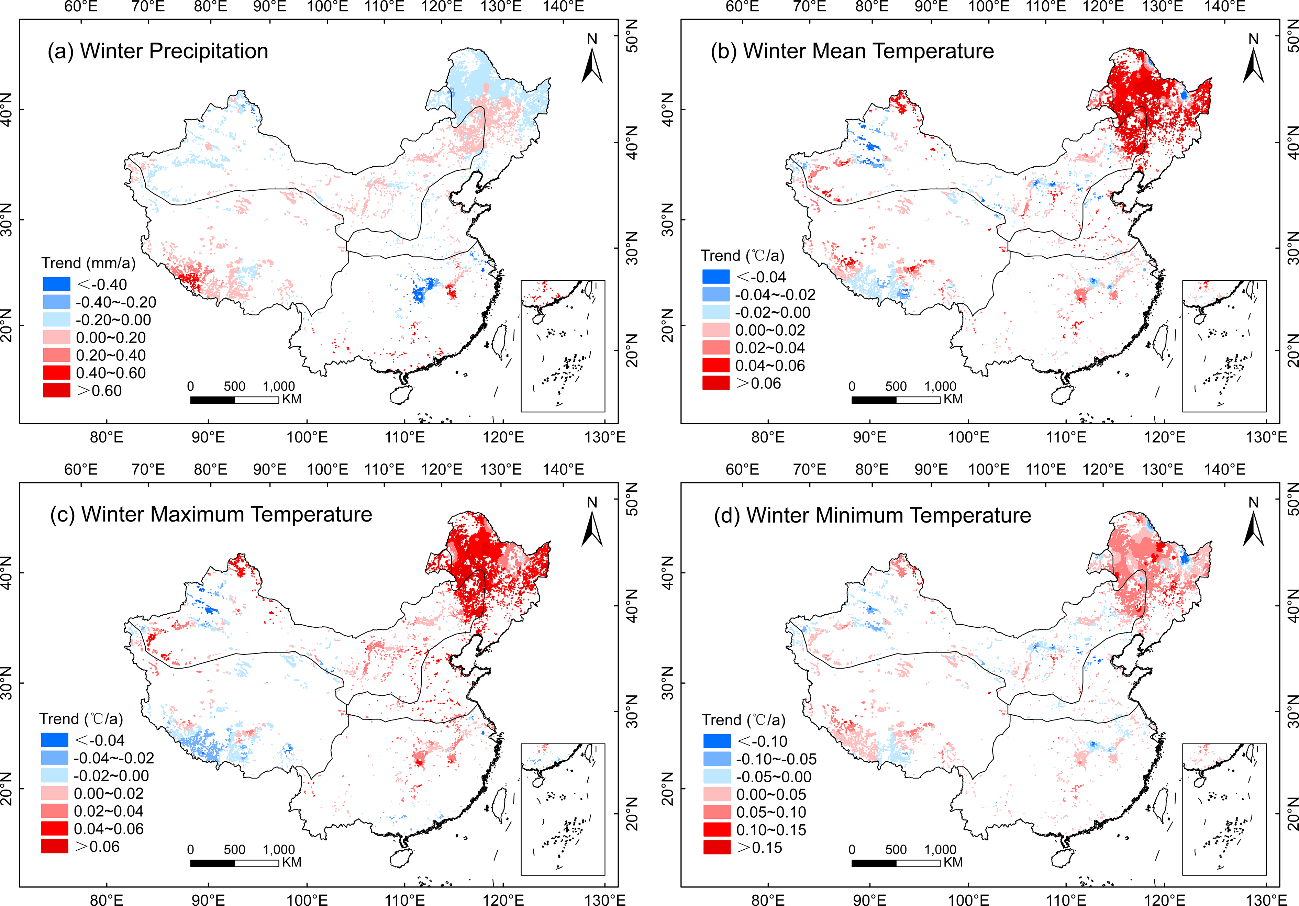


**Figure S4.** Variation trends of winter precipitation (mm/a), T_mean_ (°C/a), T_max_ (°C/a), and T_min_ (°C/a) in herbaceous marshes of China during 2000 - 2020.


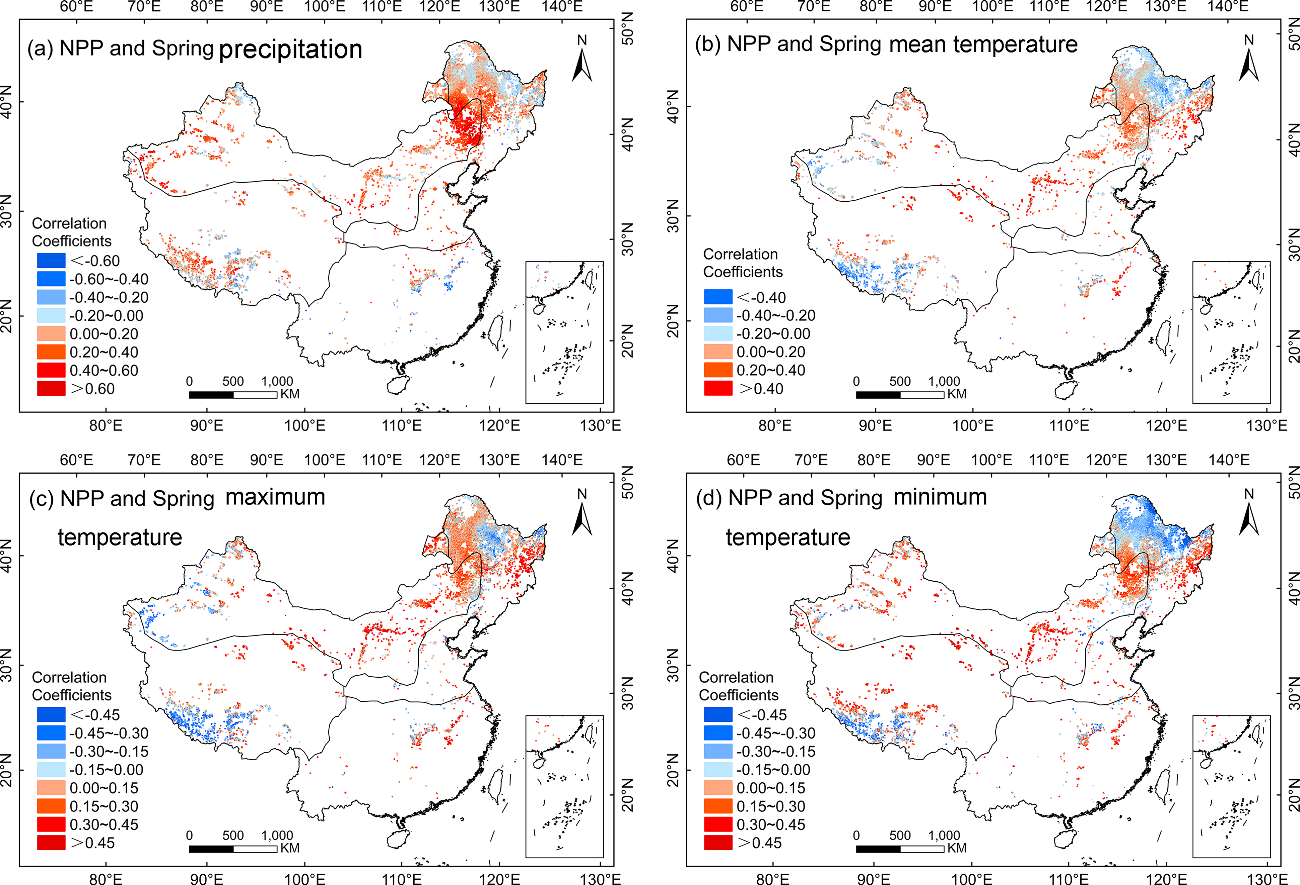


**Figure S5.** Spatial patterns in the correlation coefficients between NPP and spring climatic factors in herbaceous marshes of China during 2000 - 2020.


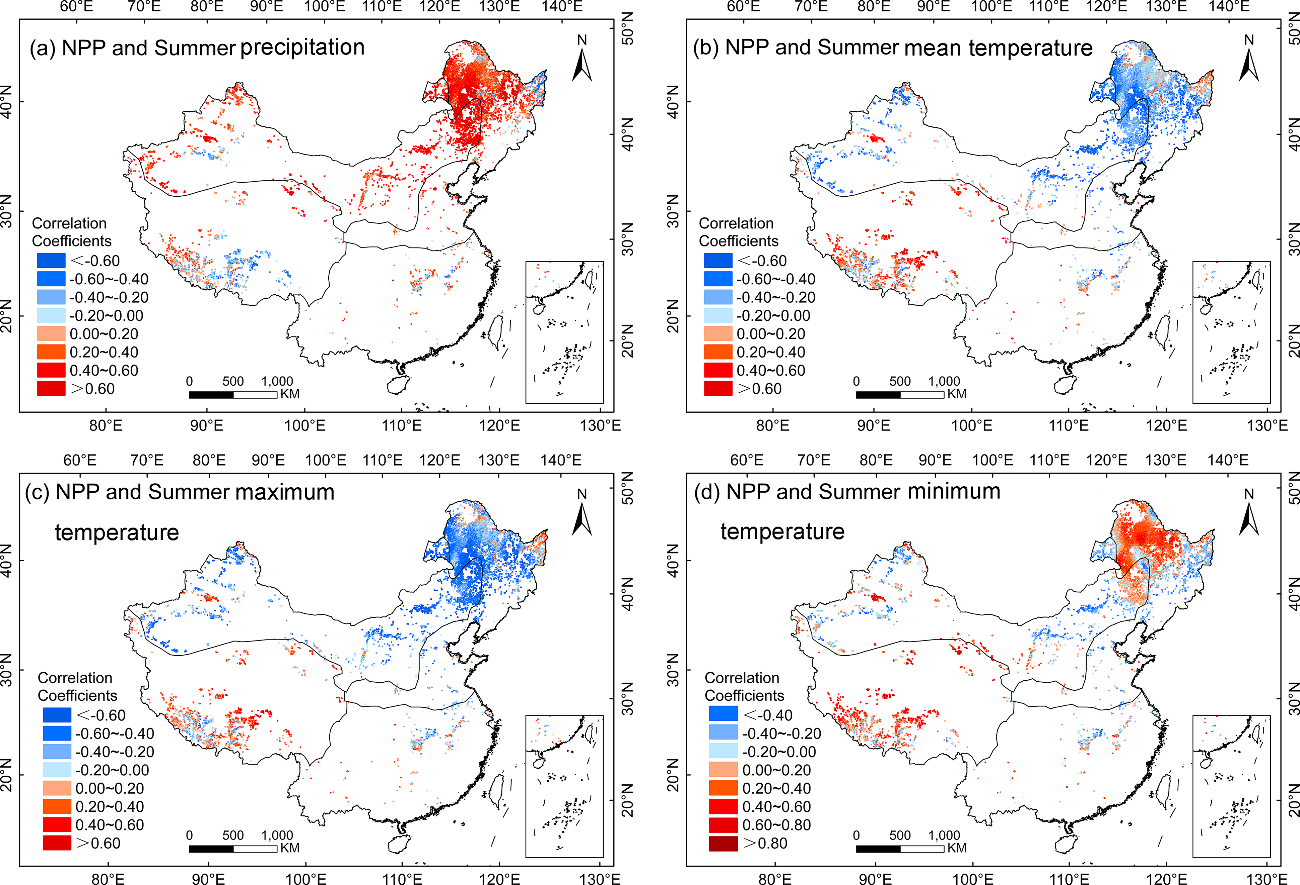


**Figure S6.** Spatial patterns in the correlation coefficients between NPP and summer climatic factors in herbaceous marshes of China during 2000 - 2020.


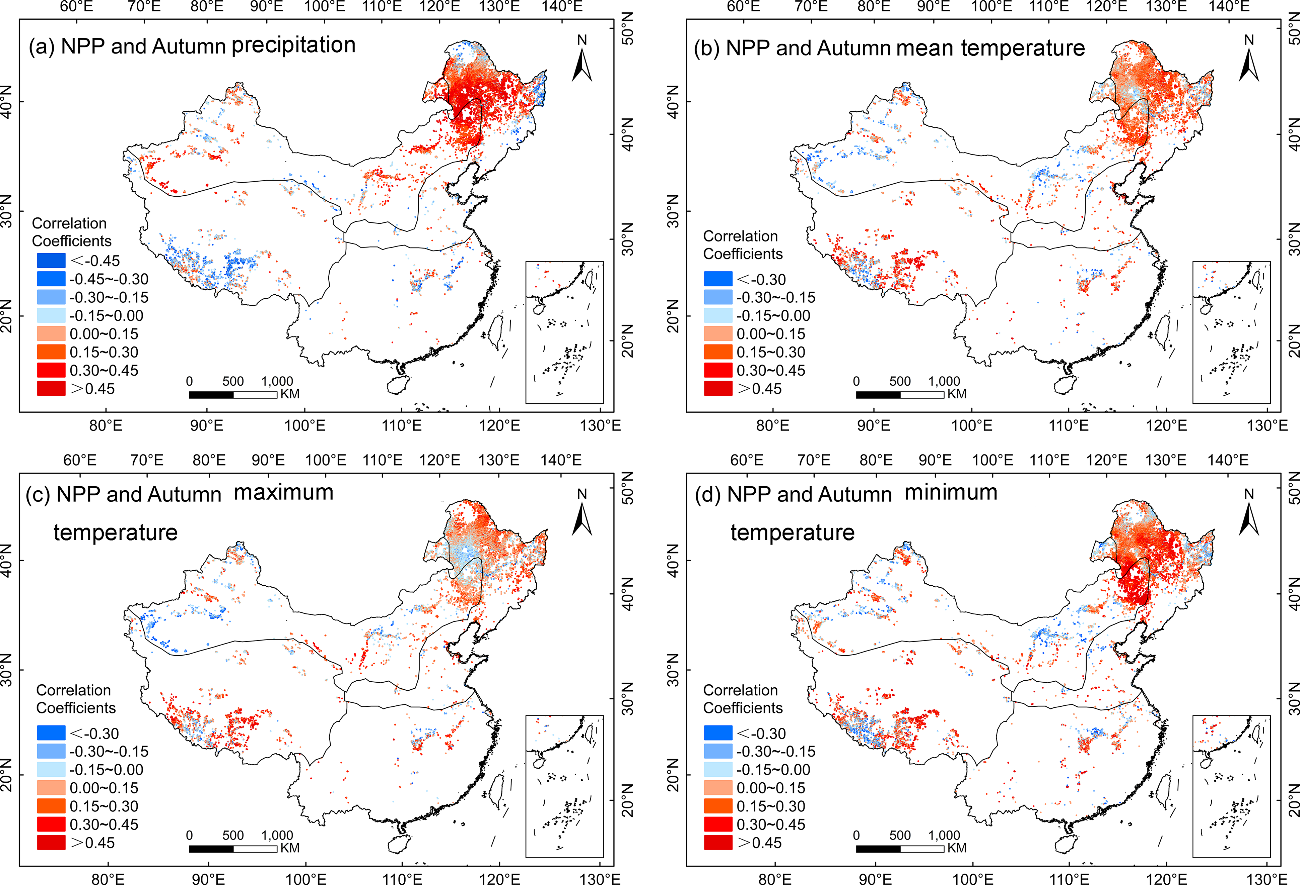


**Figure S7.** Spatial patterns in the correlation coefficients between NPP and autumn climatic factors in herbaceous marshes of China during 2000 - 2020.


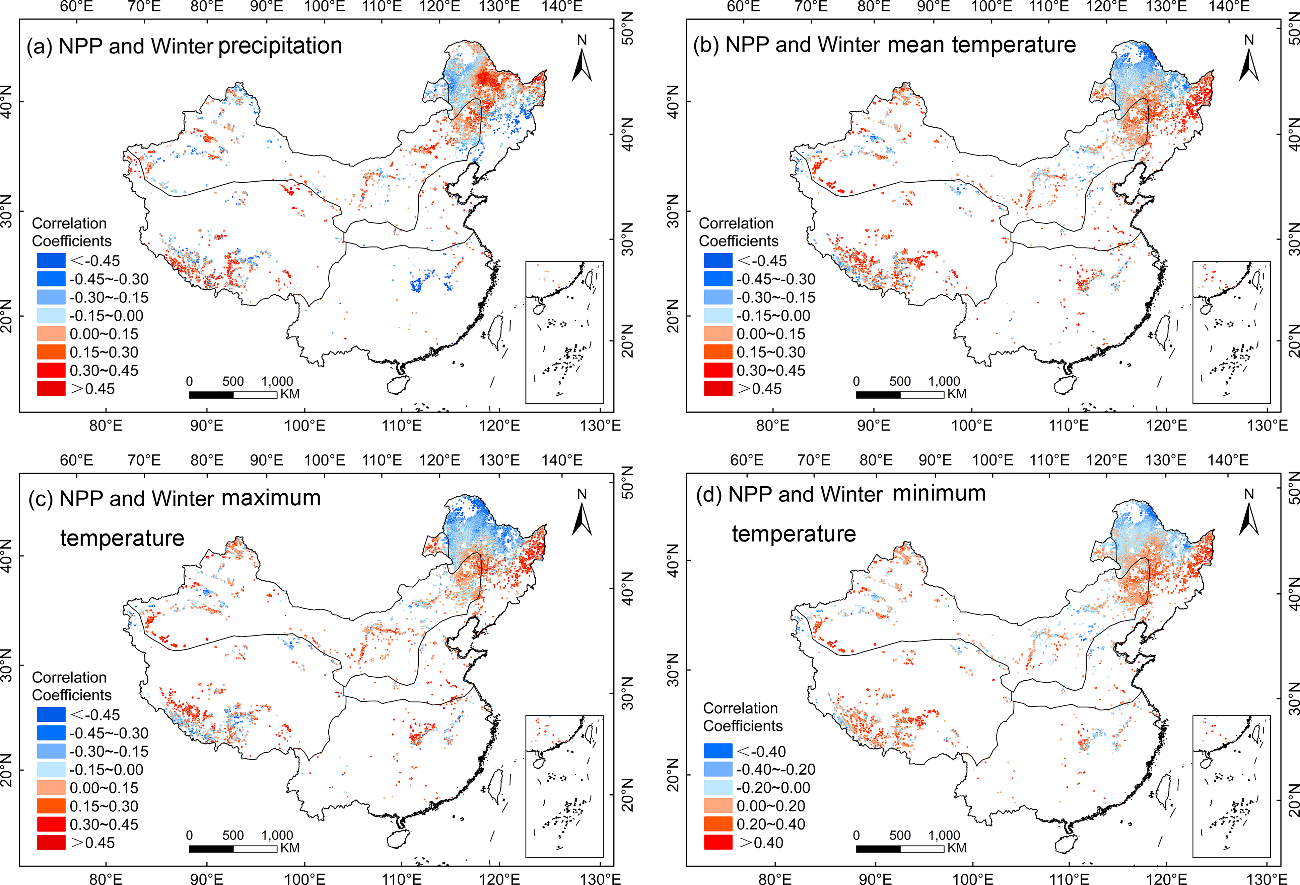


**Figure S8.** Spatial patterns in the correlation coefficients between NPP and winter climatic factors in herbaceous marshes of China during 2000 - 2020.
